# Preclinical Screening Platform Identifies Azatadine‐Dimaleate as a Potent Repurposed Therapeutic Against SARS‐CoV‐2 Infection

**DOI:** 10.1002/jmv.70713

**Published:** 2025-11-20

**Authors:** Ahlam Ali, David Courtney, Lindsay Broadbent, Parul Sharma, Connor G. G. Bamford, Sheerien Manzoor, Olivier Touzelet, Conall McCaughey, Adam Kirby, Eleanor Bentley, Anja Kipar, Ken I. Mills, James P. Stewart, Ultan F. Power

**Affiliations:** ^1^ Patrick G Johnston Centre for Cancer Research, School of Medicine, Dentistry and Biomedical Sciences Queen's University Belfast; Belfast Belfast Ireland; ^2^ Current Affiliation: School of Life Sciences University of Wolverhampton Wolverhampton UK; ^3^ Wellcome‐Wolfson Institute for Experimental Medicine, School of Medicine, Dentistry and Biomedical Sciences Queen's University Belfast; Belfast Belfast Ireland; ^4^ Department of Infection Biology and Microbiomes University of Liverpool Liverpool UK; ^5^ Regional Virology Laboratory, Belfast Trust Belfast Ireland; ^6^ Laboratory for Animal Model Pathology, Institute of Veterinary Pathology, Vetsuisse Faculty University of Zurich Zurich Switzerland

## Abstract

The emergence of SARS‐CoV‐2 posed a major global public health threat, necessitating urgent development of therapeutics. Despite vaccine availability, continuous emergence of viral variants with enhanced transmissibility and immune escape capabilities, and consequential impacts on health services, requires effective antiviral therapeutics. Drug repurposing offers an expeditious strategy to identify therapeutics with established safety profiles. We implemented a comprehensive three‐tiered validation approach, screening 2,570 compounds against SARS‐CoV‐2 in vitro, followed by *ex vivo* validation in well‐differentiated primary human bronchial epithelial cell (WD‐PBEC) cultures, and rigorous in vivo assessment. This methodical progression identified Azatadine‐Dimaleate, a H1‐receptor antagonist, as an exceptional candidate with consistent efficacy across all systems. Azatadine‐Dimaleate demonstrated potent antiviral activity‐ EC50: 4.0 µM (95% CI: 3.2–4.8 µM), reducing viral replication by ~5,000‐fold at 25 µM in epithelial cultures and lowering peak viral titers in WD‐PBECs by 1.4 log_10_, and 2.33 log_10_ at 48 and 96 hpi, respectively, compared to controls. There was also a concomitant reduction in expression of interferons and pro‐inflammatory genes, including IL‐6. Combination with Remdesivir synergistically enhanced antiviral activity, reducing the EC50 of both drugs by > 60%. In the K18‐hACE2 transgenic mouse model, Azatadine‐Dimaleate significantly reduced weight loss (4% *vs.* 12%, *p* ≤ 0.05), decreased viral loads, and halved viral antigen expression in lung tissues. Unlike many candidates that faltered in complex models, Azatadine‐Dimaleate maintained efficacy across all platforms. These findings support its clinical evaluation, alone or in combination with Remdesivir, as a versatile therapeutic with strong potential to address current and emerging SARS‐CoV‐2 variants.

## Summary

Multi‐tiered validation proves Azatadine‐Dimaleate potently inhibits SARS‐CoV‐2 with synergistic Remdesivir enhancement, offering immediate clinical potential.

## 1 Introduction

The emergence of severe acute respiratory syndrome coronavirus 2 (SARS‐CoV‐2) in December 2019 precipitated an unprecedented global health crisis that continues to present alarming challenges to healthcare systems worldwide [1]. Whilst vaccine development has substantially mitigated the immediate threat, the persistent evolution of viral variants with enhanced transmissibility and immune escape capabilities necessitates effective therapeutic interventions. This need is further accentuated by challenges in global vaccine equity, coupled with the recognition that coronaviruses represent pathogens with significant pandemic potential, evidenced by three major outbreaks within two decades.

Despite the availability of Remdesivir as an approved therapeutic, significant limitations persist in COVID‐19 treatment options. Remdesivir requires intravenous administration limiting its use to hospital settings, shows variable clinical efficacy particularly in severe disease, and remains costly with limited global availability [2]. Additionally, the emergence of resistant variants and the need for early intervention strategies necessitate identification of alternative therapeutics [3]. Ideal candidates would offer oral bioavailability enabling outpatient treatment, demonstrate synergistic activity with existing drugs to enhance efficacy and reduce resistance development, leverage established safety profiles to expedite clinical deployment, and provide cost‐effective manufacturing for global distribution, particularly in resource‐limited settings. These considerations motivated our systematic screening approach to identify repurposed drugs meeting these criteria.

Drug repurposing, the systematic investigation of existing pharmaceuticals for novel therapeutic applications offers distinct advantages over conventional *de novo* drug development pathways [4]. This approach leverages established pharmacokinetic and safety profiles, circumvents time‐consuming developmental stages, and facilitates rapid deployment during emerging infectious disease outbreaks. These strategic advantages are particularly relevant where the temporal window between pathogen identification and therapeutic necessity is compressed by exponential epidemiological dynamics.

Despite numerous drug‐screening studies identifying potential pharmaceutical candidates for COVID‐19, there continues to be a prohibitively high failure rate in translating promising *in silico* or in vitro efficacy to clinically beneficial outcomes in patients [5, 6]. This translational discontinuity stems partly from methodological limitations; primarily the reliance on simplified experimental systems that inadequately recapitulate the complex pathophysiology of SARS‐CoV‐2 infections. Such systems fail to incorporate critical aspects of respiratory epithelial architecture and functionality that modulate both viral pathogenesis and therapeutic response [7].

To address these limitations, we implemented a multi‐tiered validation strategy, progressing systematically from high‐throughput screening through *ex vivo* human airway epithelial models to in vivo assessment in a transgenic murine model of COVID‐19. This approach enables sequential validation across increasing levels of physiological complexity, identifying compounds with consistent efficacy across diverse experimental systems, a characteristic predictive of clinical translation potential.

Well‐differentiated primary bronchial epithelial cell (WD‐PBEC) cultures recapitulate the complex cellular architecture of the human respiratory epithelium, including pseudostratified organization, ciliated and secretory cell populations, and mucus production [8, 9]. These advanced systems express physiologically relevant levels of angiotensin‐converting enzyme 2 (ACE2) and transmembrane serine protease 2 (TMPRSS2), key determinants of SARS‐CoV‐2 cellular entry. Their utilization offers significant advantages in evaluating therapeutic candidates, particularly in postinfection administration scenarios that mimic clinically feasible treatment approaches.

For evaluation of therapeutic candidates against SARS‐CoV‐2, physiologically relevant in vivo systems are essential for robust preclinical validation. The K18‐human ACE2 transgenic mouse model provides a valuable platform for investigating therapeutic efficacy within a complete biological system [10]. These mice express the human ACE2 receptor under the keratin 18 promoter, enabling SARS‐CoV‐2 replication in pulmonary epithelia with development of COVID‐19‐like pathological changes [11]. This model permits assessment of virological parameters and disease manifestations that cellular systems cannot adequately recapitulate, including immune cell infiltration and systemic responses. Integrating animal models within our validation framework provides crucial evidence of therapeutic efficacy in physiological contexts approximating human disease.

Herein, we describe a comprehensive evaluation of Azatadine‐Dimaleate, a H1‐receptor antagonist identified through systematic screening of 2,570 compounds with established human safety profiles. We validated this compound's efficacy across increasingly complex experimental systems, culminating in demonstration of significant therapeutic benefits in the K18‐human ACE2 transgenic mouse model of COVID‐19. The consistency of Azatadine‐Dimaleate's efficacy across diverse models strongly suggests translational potential worthy of clinical evaluation. Furthermore, it's demonstrated synergistic interaction with Remdesivir offers considerable potential for combination strategies that might simultaneously mitigate resistance development whilst maximizing antiviral efficacy.

Our comprehensive preclinical findings provide robust evidence supporting the utility of Azatadine‐Dimaleate, either as monotherapy or in strategic combination regimens, as a valuable contribution to the therapeutic armamentarium against both current SARS‐CoV‐2 variants and potential future coronavirus threats.

## 2 Results

### 2.1 High Throughput Screening Assay Development

**Figure 1:**
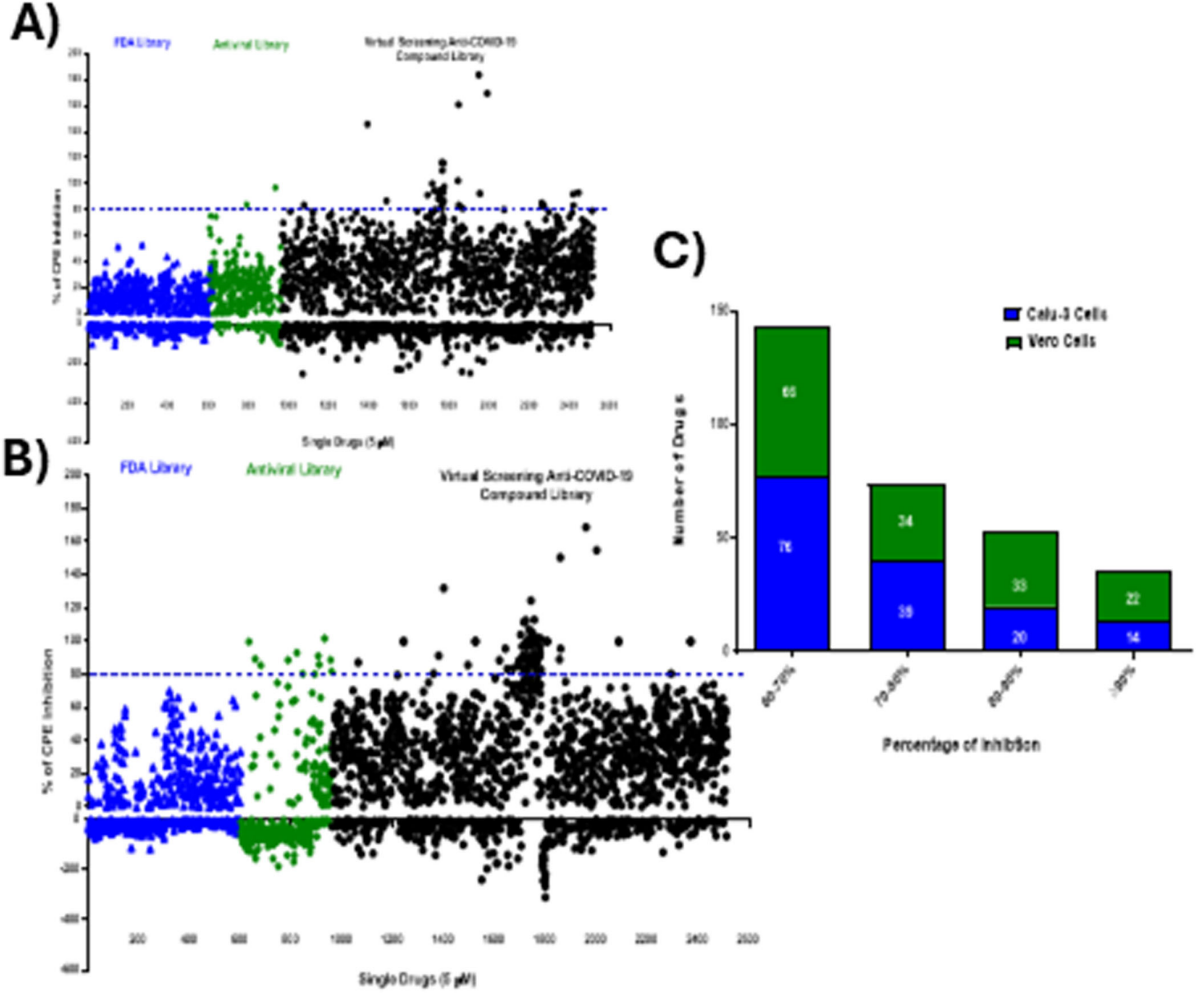
Systematic high‐throughput screening identifies potential SARS‐CoV‐2 inhibitors. Single agent screen of 2,570 drugs in Calu‐3 (A) and Vero cells (B). Drugs were added at a concentration of 5 µM and cells were incubated for 24 h, followed by SARS‐COV‐2 infection at Multiplicity of Infection (MOI) = 0.1. At 72 hpi, CPE was measured using CellTox™ Green reagent by the Clariostar plus plate reader. Each assay plate contained control wells with mock infected cells, and control wells with cells infected with SARS‐COV‐2. All control wells were treated with dimethyl sulfoxide (DMSO) at the same concentration as assay wells and used to calculate a Z'‐value for each plate and to normalize the data on a per plate basis. Results were expressed as a percentage inhibition of CPE where 100% inhibition of CPE was equal to the mean of the mock infected cell controls, and 0% of inhibition was equal to the mean of the SARS‐COV‐2 infected cell controls. Each data point represents an average of two separate repeat experiments. C) Number of potential hits for both Vero and Calu‐3 cells from the primary screen.

We screened a panel of human and simian cell lines (Figure S1) to identify cells that are permissive to SARS‐CoV‐2 infection. Vero, Vero‐E6, Huh7, and Calu‐3 cells were highly susceptible to infection, producing obvious cytopathic effect (CPE) and were extensively used as model systems to screen for antivirals [12, 13, 14]. For drug screening purposes, we chose both Calu‐3 and Vero cells. Drug candidates were tested using an in vitro CPE‐based assay (Figure S2‐B), which was successfully adapted into high throughput format. Data were normalized to the median of each plate and used to calculate the Log_2_ fold change (Figure S2‐C), which enabled us to establish a reliable dynamic range based on the activity of a positive control (Remdesivir 1 μM). The correlation coefficient (R^2^) for the two replicates was determined to be 0.7431 for both Vero and Calu‐3 cells (Figure S2‐D). The reproducibility and robustness of the duplicate screens were demonstrated with similar Z' values (0.76 vs 0.79). Hits were selected based on demonstration of ≥ 80% reduction in CPE in infected cells per well while also having > 90% cell viability (Figure 1‐A & B, Fig S3).

### 2.2 High‐Throughput Screen Identified Multiple Drugs Effective Against SARS‐CoV‐2

**Figure 2:**
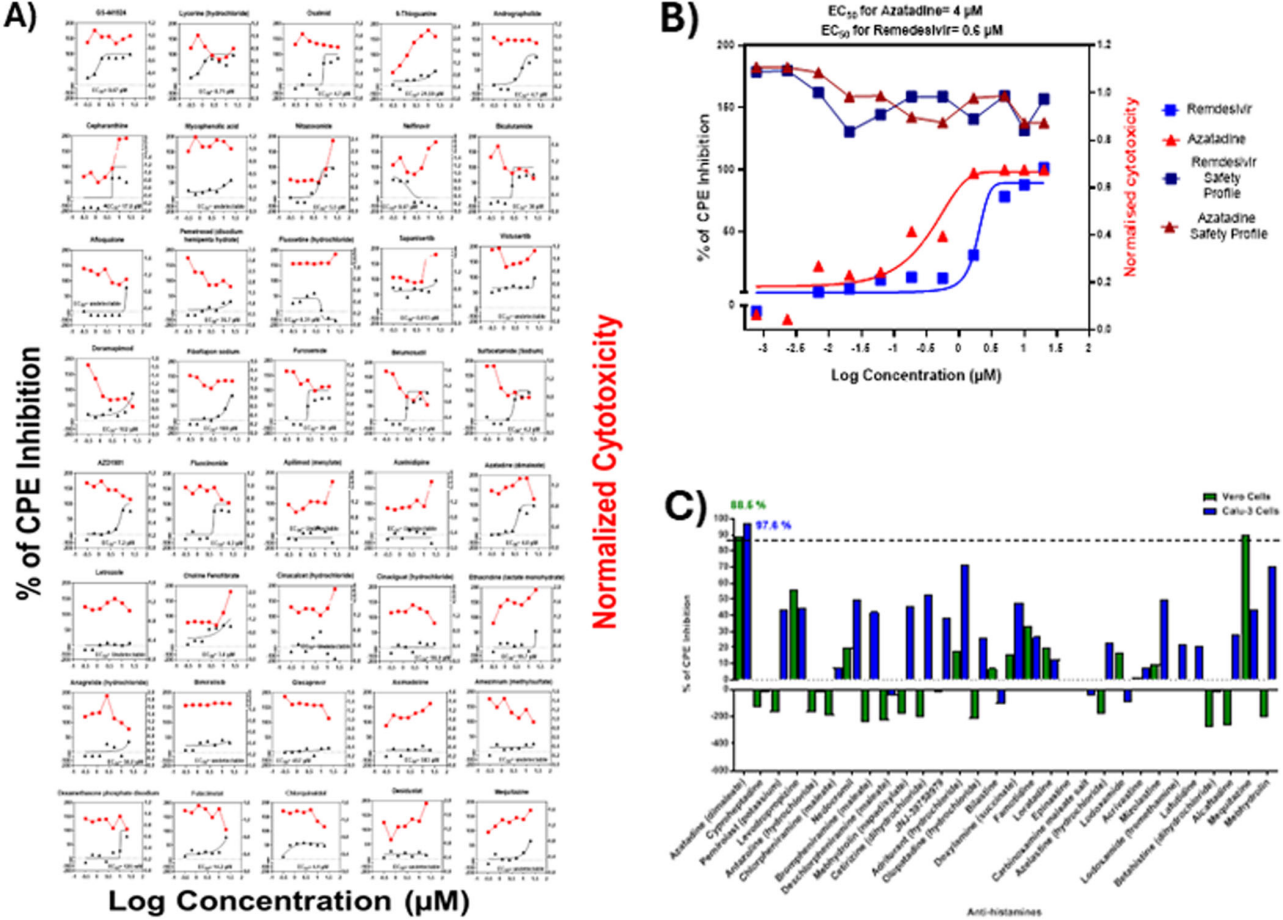
Validation of hits identifies Azatadine‐Dimaleate as a potent SARS‐CoV‐2 inhibitor. (A) Validation of the selected 40 single molecules identified in the initial drug screen. Each candidate underwent 7 serial dilutions of 1 in 2, starting at 20 µM to test their ability to diminish cytotoxicity in SARS‐CoV‐2‐infected Calu‐3 cells (PHE MOI = 0.1) at 72 hpi. Three days post infection CPE was measured as per Fig.‐1. Data is representative of the average of three separate repeat experiments. (B) Validation of antiviral activity of Azatadine‐Dimaleate in a 96 well plate format. Remdesivir and Azatadine‐Dimaleate underwent 7 serial dilutions of 1 in 2, from a starting concentration of 20 µM to test their ability to prevent cytotoxicity in SARS‐CoV‐2‐infected Calu‐3 cells (MOI = 0.1) at 72 hpi and EC50s with 95% confidence intervals were calculated using nonlinear regression analysis. Cytotoxicity profiles of the drugs were determined in parallel. (C) Single agent screen of 29 antihistamine drugs in Calu3 and Vero cells. Drugs were added at a concentration of 5 µM and cells were incubated for 24 h, followed by SARS‐COV‐2 infection (PHE MOI = 0.1). At 72 hpi, CPE was measured as per Fig.‐1. Data represents an average of 3 separate repeat experiments.

In our primary screen, there were 33 drugs in Vero cells and 20 drugs in Calu‐3 cells that showed efficacy between 80% and 90% in the CPE inhibition assay (Figure. 1‐C), with five drugs overlapping in both cell lines. These latter hits included Sapanisertib, Lycorine (hydrochloride), Glecaprevir, GS‐441524, and Azatadine‐Dimaleate. Notably, certain compounds demonstrated pronounced cell‐type specificity in their antiviral activity profiles. Apilimod (mesylate), Chloroquine and hydroxychloroquine, for instance, demonstrated 100% CPE inhibition in Vero cells but showed negligible antiviral activity in Calu‐3 cells, suggesting potential cell‐ or species‐specific mechanisms of action or differential expression of target proteins. This observation underscores the value of employing multiple cell lines for antiviral screening to identify compounds with consistent efficacy across diverse cellular contexts. From our comprehensive analysis of the screening data, we selected 40 drugs for further validation alongside Remdesivir as a positive control (Figure. S3). Selection criteria incorporated both single‐cell efficacy ( ≥ 80% inhibition of SARS‐CoV‐2‐induced CPE in one cell line) and cross‐platform activity ( ≥ 75% inhibition of SARS‐CoV‐2 induced CPE in both cell lines) (Table S2; Figure. 2‐A).

### 2.3 Validation of Hits From Primary Screen Revealed an Effective Drug Against SARS‐CoV‐2

Our CPE‐based screening detected cytoprotection rather than directly measuring viral replication, potentially yielding false positives through cell‐protective mechanisms independent of antiviral activity. To address this limitation, all primary screening hits underwent rigorous secondary validation using orthogonal assays, including plaque assays for infectious virus quantification and RT‐qPCR for viral RNA measurement, ensuring that only compounds with genuine antiviral activity were advanced for further study. All validation studies were conducted in human lung epithelial Calu‐3 cells in 96‐well plates, providing a physiologically relevant context for assessing potential COVID‐19 therapeutics. Following rigorous validation, 28 of the initial 40 compounds were eliminated due to insufficient inhibition of SARS‐CoV‐2‐induced CPE (40%–60% CPE inhibition) under these more stringent experimental conditions. Furthermore, 9/40 drugs demonstrated efficacy against SARS‐CoV‐2‐induced CPE, but only at higher concentrations associated with elevated EC50 values (≥ 10 µM) and concerning toxicity profiles, limiting their therapeutic potential.

Most significantly, 3/40 drugs exhibited exceptional antiviral properties, characterized by 100% efficacy at low EC50 values (0.67–4 µM). Lycorine hydrochloride [15] (EC50 = 0.67 µM, 95% CI: 0.5–0.9 µM) and GS‐441524 [16] (EC50 = 0.85 µM, 95% CI: 0.6–1.1 µM), were previously documented as SARS‐CoV‐2 inhibitors, providing important internal validation of our screening methodology. The third potent antiviral compound identified was Azatadine‐Dimaleate, an antihistamine that demonstrated Remdesivir‐like CPE inhibition with an EC50 of 4.0 µM (95% CI: 3.2–4.8 µM) (Figure. 2‐A and B). Notably, amongst the 30 antihistamines represented in our screening library, Azatadine‐Dimaleate demonstrated unique antiviral properties, with structurally related compounds, including Loratadine, exhibiting negligible activity against SARS‐CoV‐2. The exception was Mequitazine, which showed modest efficacy in Vero cells only (Figure. 2‐C). This distinctive pharmacological profile suggests that Azatadine‐Dimaleate's antiviral activity may be mediated through mechanisms distinct from its canonical H1‐receptor antagonism.

### 2.4 Validation of Hits From Primary Screen Revealed Azatadine‐Dimaleate as an Effective Drug Against SARS‐CoV‐2 With Synergistic Effects When Combined With Remdesivir

**Figure 3:**
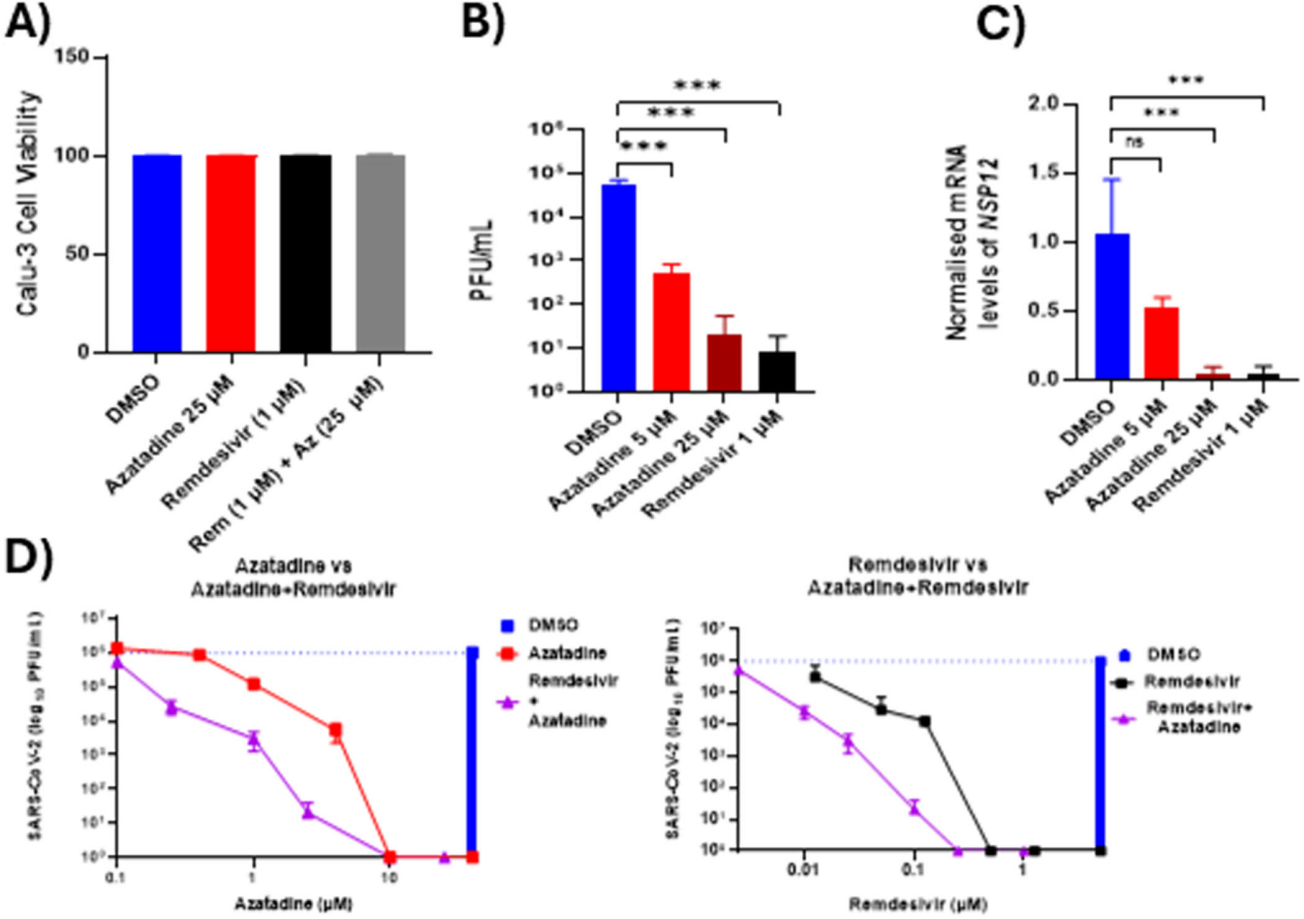
Azatadine‐Dimaleate exhibits dose‐dependent antiviral activity and synergistic enhancement with Remdesivir. (A) Cell viability assessment in uninfected Calu‐3 cells treated with DMSO control, Azatadine‐Dimaleate (25 µM), Remdesivir (1 µM), or the combination (Remdesivir 1 µM + Azatadine 25 µM) for 72 h. No cytotoxic effects were observed with any treatment. (B) Release of infectious virus was quantified by plaque assay. Calu‐3 cells were pretreated with Azatadine‐Dimaleate (5 & 25 µM) or Remdesivir (1 µM) 24 h before infection with SARS‐CoV‐2 PHE (MOI = 0.1). (C) Levels of viral NSP12 RNA was measured by RT‐qPCR following treatment with Azatadine‐Dimaleate at (5 & 25 µM) or Remdesivir (1 µM) 24 h before infection with SARS‐CoV‐2 (PHE MOI = 0.1). Data bars represent mean ± SEM of n = 3 independent experiments. Statistical analysis by one‐way ANOVA, ***p ≤ 0.001 vs DMSO control. (D) A concentration gradient of Remdesivir, Azatadine‐Dimaleate, or combined drugs was used to determine EC50s with 95% confidence intervals for these conditions in infected Calu‐3 cells pretreated 24 h before infection (PHE MOI = 0.1), with either Remdesivir (starting at 5 µM), Azatadine‐Dimaleate (starting at 40 µM), or a combination of the two with Remdesivir (starting at 1 µM) and Azatadine‐Dimaleate (starting at 25 µM). DMSO alone was used as a negative control. At 72 hpi viral titres were determined by plaque assay. Data points represent an average of 3 separate repeat experiments ±SEM.

Further validation of the effectiveness of Azatadine‐Dimaleate using standard molecular virology techniques demonstrated its potent antiviral properties. Cytotoxicity assessment confirmed that Azatadine‐Dimaleate alone (25 µM) or in combination with Remdesivir (1 µM) exhibited no discernible toxicity on Calu‐3 cells (Figure. 3A), indicating a favourable therapeutic index. Quantification of infectious virus production by plaque assay following pretreatment of Calu‐3 cells with Azatadine‐Dimaleate (5 & 25 µM) or Remdesivir (1 µM) demonstrated dose‐dependent inhibition of viral replication. Azatadine‐Dimaleate treatment resulted in approximately 100‐fold and 5,000‐fold reductions in viral titres at 5 and 25 µM, respectively, while 1 µM Remdesivir demonstrated comparable inhibitory activity to 25 µM Azatadine‐Dimaleate (Figure 3B). Complementary molecular analysis by RT‐qPCR revealed that 5 µM Azatadine‐Dimaleate reduced viral *NSP12* RNA levels by approximately 50% relative to untreated controls, while 25 µM Azatadine‐Dimaleate or 1 µM Remdesivir suppressed *NSP12* RNA levels by > 95% (Figure. 3C), confirming potent inhibition of viral replication.

We proceeded to test whether Azatadine‐Dimaleate could be used in combination with Remdesivir (Figure. 3D). Remdesivir alone and Azatadine‐Dimaleate alone had EC50s of 0.6 µM (95% CI: 0.4–0.8 µM) and 4.0 µM (95% CI: 3.2–4.8 µM), respectively. However, in combination, Remdesivir and Azatadine‐Dimaleate had EC50 values of 0.25 µM (95% CI: 0.15–0.35 µM) and 2.0 µM (95% CI: 1.5–2.5 µM), respectively. This constituted a strong reduction in concentrations of both drugs required to inhibit viral replication, representing a 2.4‐fold and twofold improvement in potency for Remdesivir and Azatadine‐Dimaleate, respectively. The enhanced antiviral efficacy demonstrated a therapeutic synergistic effect between Remdesivir and Azatadine, as confirmed by the Combination index (CI) determined by Compusyn program (CI = 0.034). This CI value substantially below 1.0 indicates potent pharmacological synergy, suggesting these compounds may target distinct yet complementary aspects of the viral replication cycle.

### 2.5 Validation of Azatadine‐Dimaleate Therapeutic Potential in Primary Bronchial Epithelial Cells

**Figure 4:**
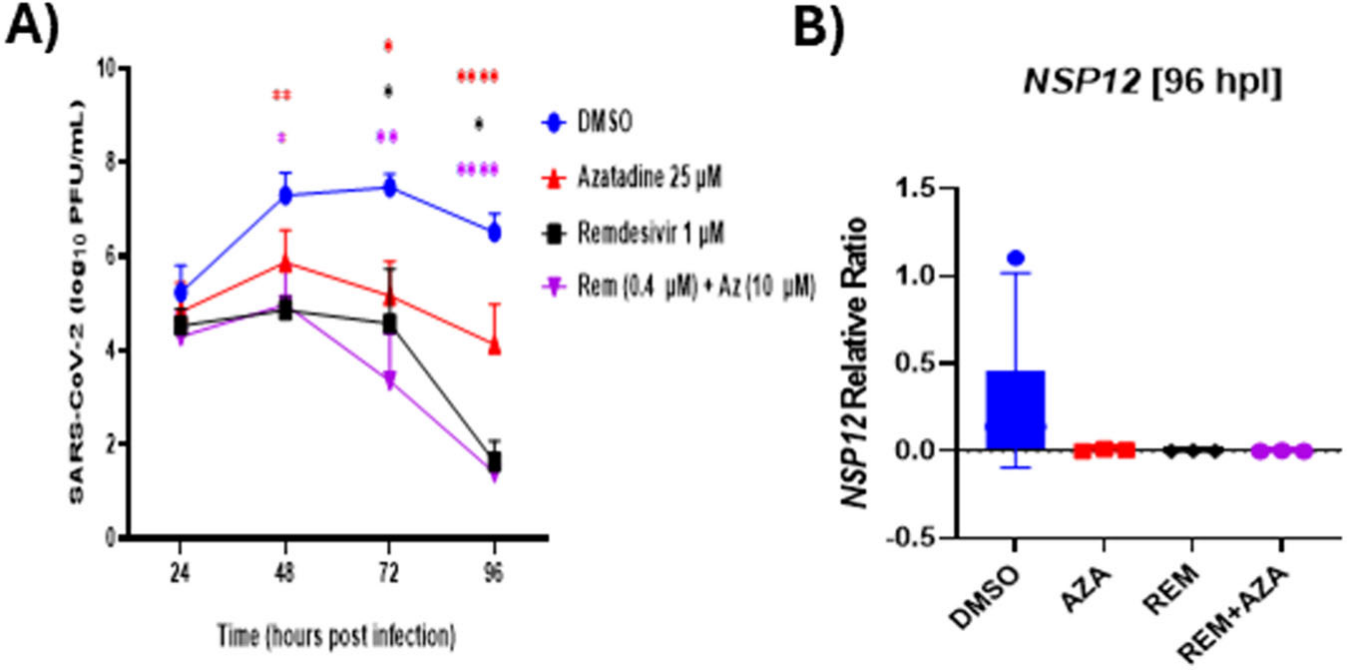
Azatadine‐Dimaleate inhibits SARS‐CoV‐2 replication in well‐differentiated primary bronchial epithelial cells. (A) WD‐PBECs from 5 donors were infected with SARS‐CoV‐2 (MOI = 1). At 24 hpi cultures were rinsed apically to titrate released virus and treated with Remdesivir (1 µM) or Azatadine‐Dimaleate (25 µM) alone, or a combination of both (0.4 µM Remdesivir and 10 µM Azatadine‐Dimaleate) or DMSO, and freshly treated every 24 h thereafter. Apical rinses were repeated at 48, 72 and 96 hpi and virus titers determined by plaque assay. (B) Inhibition of viral NSP12 gene expression in SARS‐CoV‐2‐infected WD‐PBECs following Azatadine‐Dimaleate +/‐ Remdesivir treatment. RNA samples were harvested at 96 hpi from each condition in duplicate from WD‐PBECs and NSP12 RNA expression was analyzed using RT‐qPCR. The relative ratios were calculated in reference to a house keeping gene GAPDH using light cycler 96 software 1.1. The data value plotted for each donor in each condition is an average of 2 biological duplicate Transwells and 4 technical replicates.

Significant reductions in SARS‐CoV‐2 growth kinetics in WD‐PBECs were evident following all antiviral treatments compared to DMSO. Remdesivir and the combination treatment resulted in the greatest reduction in viral titers and was significant at each time point following treatment initiation (48, 72, and 96 hpi [24–72 h post treatment]). Azatadine‐Dimaleate significantly reduced viral titers at 72 and 96 hpi. All treatments prevented mean viral titers exceeding 10^6^ PFU/mL, whereas DMSO control cultures resulted in a peak viral titer of 10^7.4^ PFU/mL at 72 hpi (Figure. 4‐A). This inhibition represents a clinically relevant reduction of more than 25‐fold in viral replication that was maintained throughout the experimental time course. Validation of the antiviral potential of Azatadine‐Dimaleate by SARS‐CoV‐2 NSP12 RNA quantification in total intracellular RNA confirmed these results, demonstrating a substantial reduction (20‐fold change) in viral RNA in all treatment groups compared to controls (Figure. 4‐B).

**Figure 5:**
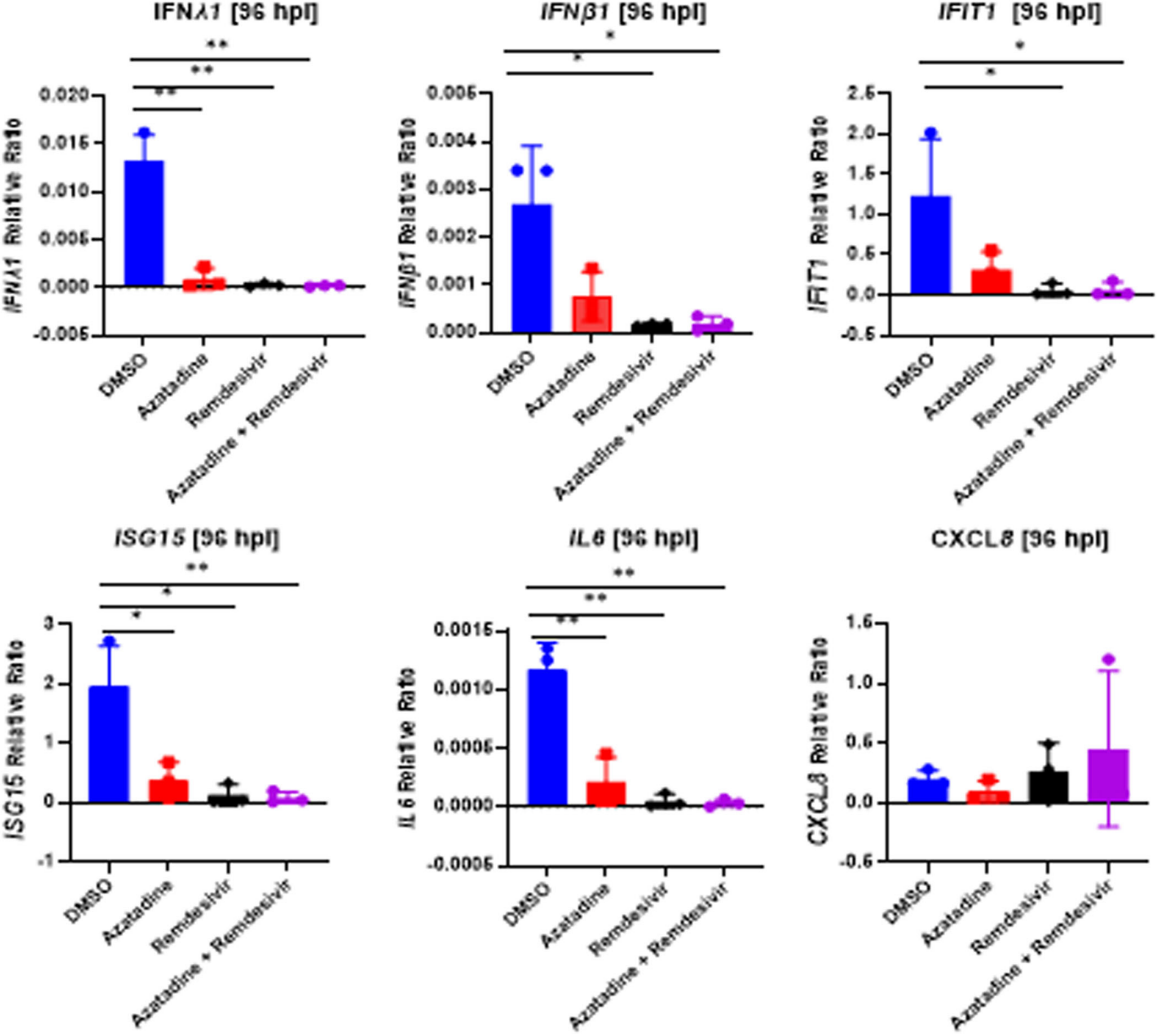
Azatadine‐Dimaleate modulates innate immune responses in WD‐PBEC cultures. RNA samples were harvested at 96 hpi from each condition in duplicate from WD‐PBECs and a panel of selected innate immune genes (IFNL1, IFNB, IFIT1, ISG15, IL‐6, CXCL8/IL8), were quantified using RT‐qPCR assay. The relative ratios for each gene were calculated in reference to a house keeping gene GAPDH using light cycler 96 software 1.1. The data value plotted for each donor in each condition is an average of 2 biological duplicate Transwells and four technical replicates. Where possible, statistical significance was determined using unpaired Student t‐test between DMSO control and individual treatments (*p ≤ 0.05, **p ≤ 0.01).

Additionally, RT‐qPCR quantification of the expression of selected innate immune response genes (*IFNL1*, *IFNB*, *IFIT1*, *ISG15*, *IL6*, *CXCL8/IL8*) demonstrated significant reductions in all except *CXCL8/IL8*, despite drug treatment initiation at 24 hpi (Figure. 5). Of note, *CXCL8/IL8* is constitutively expressed at very high levels in WD‐PBECs, which may explain the lack of observable treatment effect on this chemokine. The broad suppression of pro‐inflammatory cytokines and antiviral interferons suggests that Azatadine‐Dimaleate not only inhibits viral replication but may also have immunomodulatory effects that could be relevant in the context of COVID‐19, though it is important to note that these observations are based on respiratory epithelial cells rather than alveolar epithelial cells, which are the primary site of pathology in severe COVID‐19 lung disease.

### 2.6 Azatadine‐Dimaleate Has Therapeutic Potential in a K18‐hACE2 Mouse Model of COVID‐19

To further test the potential of Azatadine‐Dimaleate as a therapeutic against the pulmonary effects of SARS‐CoV‐2, an established preclinical mouse model of COVID‐19 was utilized. Here, K18‐hACE2 mice, which express the human ACE2 receptor under the control of the keratin 18 promoter [17], were treated intraperitoneally (i.p.) with Azatadine‐Dimaleate (10 mg/kg) and then 2 h later were challenged with 10^4^ PFU SARS‐CoV‐2 LIV strain via the intranasal route. The dose of azatadine was repeated every 24 h. The effect of Azatadine‐Dimaleate was compared with vehicle alone (DMSO), remdesivir (in the form of metabolite GS‐441524; 25 mg/kg daily) [18] and azatadine in combination with remdesivir. The dose of Azatadine‐Dimaleate was approximately 8× the ED₅₀ of histamine inhibition in mice [19] and that of remdesivir was known to be effective against SARS‐CoV‐2 infection in vivo [20]. The weight of the mice was monitored every 24 h as a noninvasive clinical sign of disease. Four days postinfection, mice were euthanized and lung tissues collected and analyzed for viral load by qRT‐PCR and virus titers, as well as histopathological changes and viral antigen expression.

**Figure 6:**
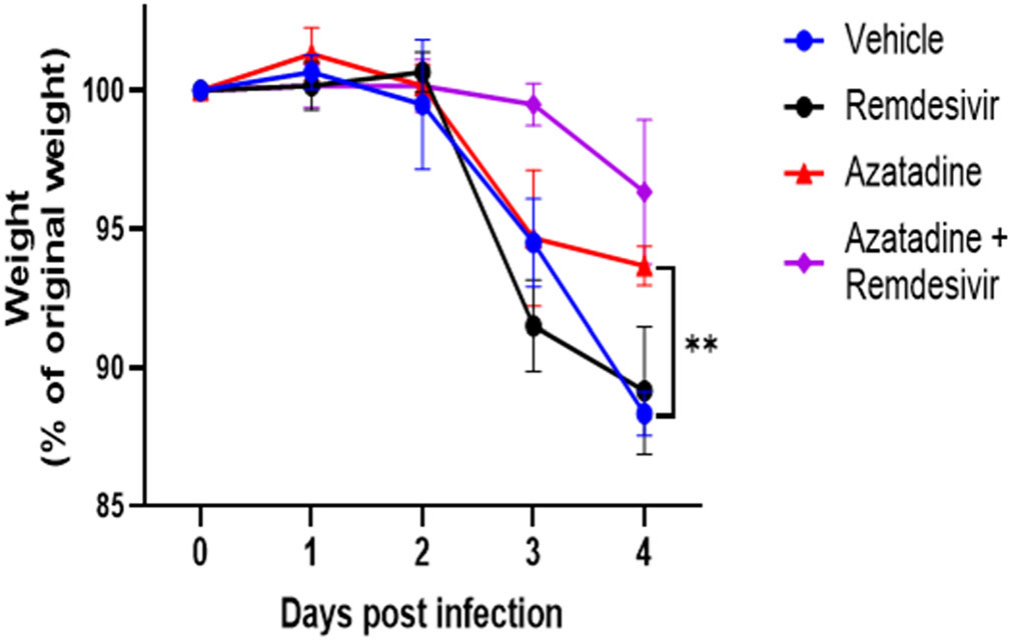
Azatadine‐Dimaleate reduces disease severity in the K18‐hACE2 transgenic mouse model. Percentage weight change of each treatment group throughout the entire study duration. K18‐hACE2 mice in each treatment group (*n* = 6) were weighed every day from day 0 to day 4. Weights are represented as a percentage of the initial weight measured at the beginning of the study. Two‐way ANOVA multiple comparison with Bonferroni correction was used to determine statistical significance. *= *p* ≤ 0.05, **= *p* ≤ 0.01.

Weight monitoring, a well‐established noninvasive indicator of disease severity, revealed distinct treatment‐dependent patterns (Figure. 6). Vehicle‐treated infected animals exhibited weight loss after day 2, reaching a mean 12% reduction by day 4. Notably, mice receiving Azatadine‐Dimaleate showed significantly reduced weight loss (mean 4%, *p* ≤ 0.05) compared to vehicle controls, indicating substantial disease attenuation. Animals treated with combination therapy demonstrated similar weight preservation (mean 4% reduction), although this did not reach statistical significance in the final timepoint analysis. Further analysis at day 3 postinfection revealed that the combination therapy showed a trend towards significance (*p* = 0.07) compared to vehicle controls. Statistical comparisons were performed at specific timepoints (not as area under the curve) using two‐way ANOVA with Bonferroni correction, as detailed in Figure 6. Interestingly, remdesivir monotherapy failed to prevent significant weight loss, with these animals showing weight trajectories similar to vehicle controls.

**Figure 7:**
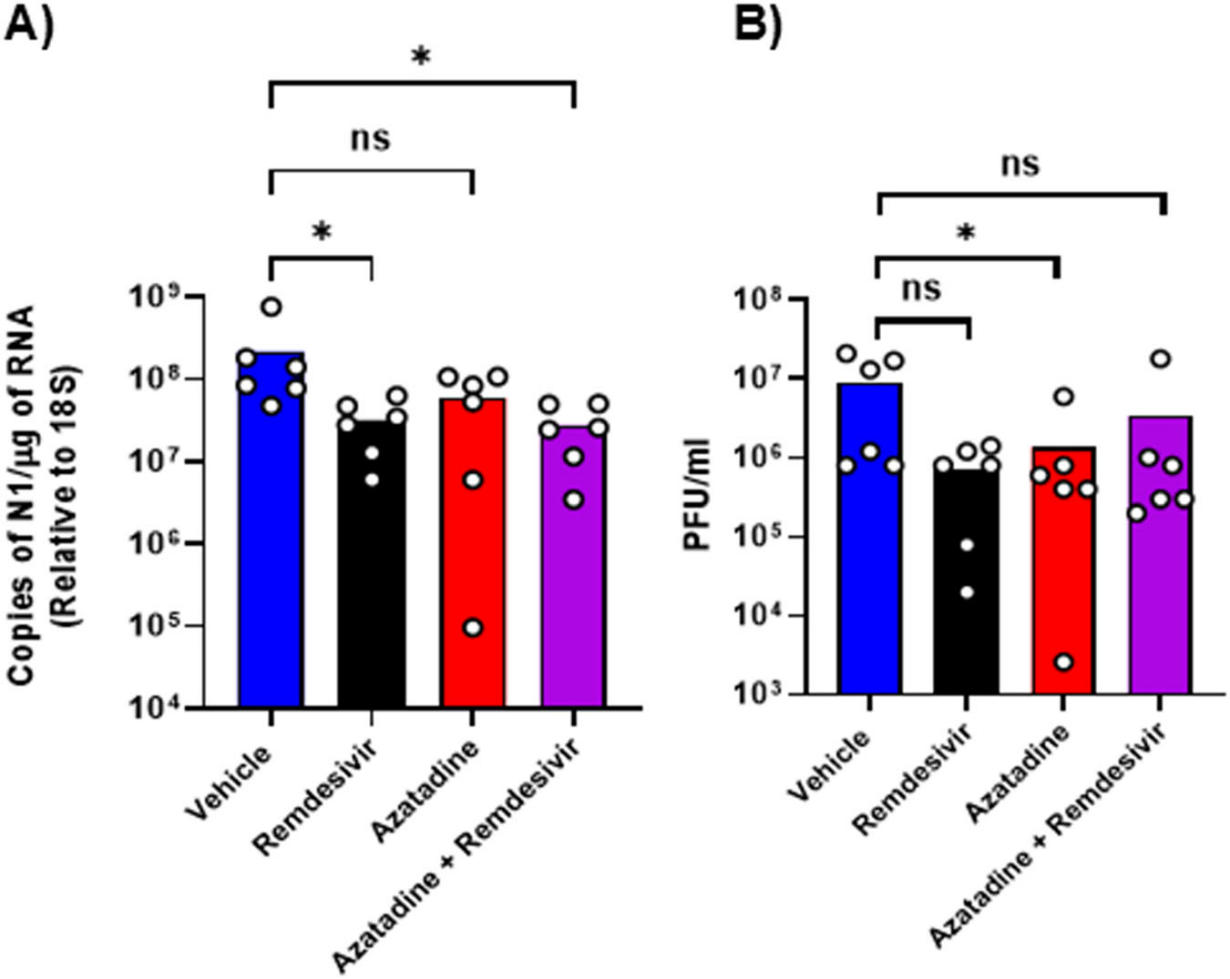
Azatadine‐Dimaleate reduces viral load in infected lung tissue. Viral RNA loads and virus titers. K18‐hACE2 mice (*n* = 6) were intranasally challenged with SARS‐CoV‐2 LIV strain at a dose of 10^4^ PFU SARS‐CoV‐2, treated with compounds or vehicle alone as indicated, and examined at day 4 post infection. (A). RNA extracted from lungs was analyzed for virus RNA levels by qRT‐PCR. Assays were normalized relative to the levels of cellular 18S RNA. **(B)** SARS‐CoV‐2 viral titers were determined using plaque assay. Data for individual animals are shown with the mean value represented by a column. Comparisons were made using one‐way ANOVA with Dunnett's multiple comparison test *Represents *p* ≤ 0.05.

Virological assessment through qRT‐PCR quantification of pulmonary viral RNA loads (Figure. 7A) revealed that all treatment regimens reduced mean viral burden compared to vehicle controls. This reduction achieved statistical significance in remdesivir and combination therapy groups (*p* ≤ 0.05). Analysis of infectious virus by plaque assay (Figure. 7B) demonstrated a similar pattern, with statistical significance reached in the Azatadine‐Dimaleate monotherapy group (*p* ≤ 0.05). These findings indicate that Azatadine‐Dimaleate effectively suppresses SARS‐CoV‐2 replication in vivo, consistent with our in vitro observations. It is noteworthy that in the K18‐hACE2 mouse model, viral antigen expression is restricted to alveolar epithelial cells, with no detectable viral antigen in lower airway epithelial cells [21]. This differs from humans where SARS‐CoV‐2 also infects the pulmonary respiratory epithelium, which is an important consideration when interpreting results across different model systems.

### 2.7 Azatadine‐Dimaleate Has Therapeutic Potential in a K18‐hACE2 Mouse Model of COVID‐19

**Figure 8:**
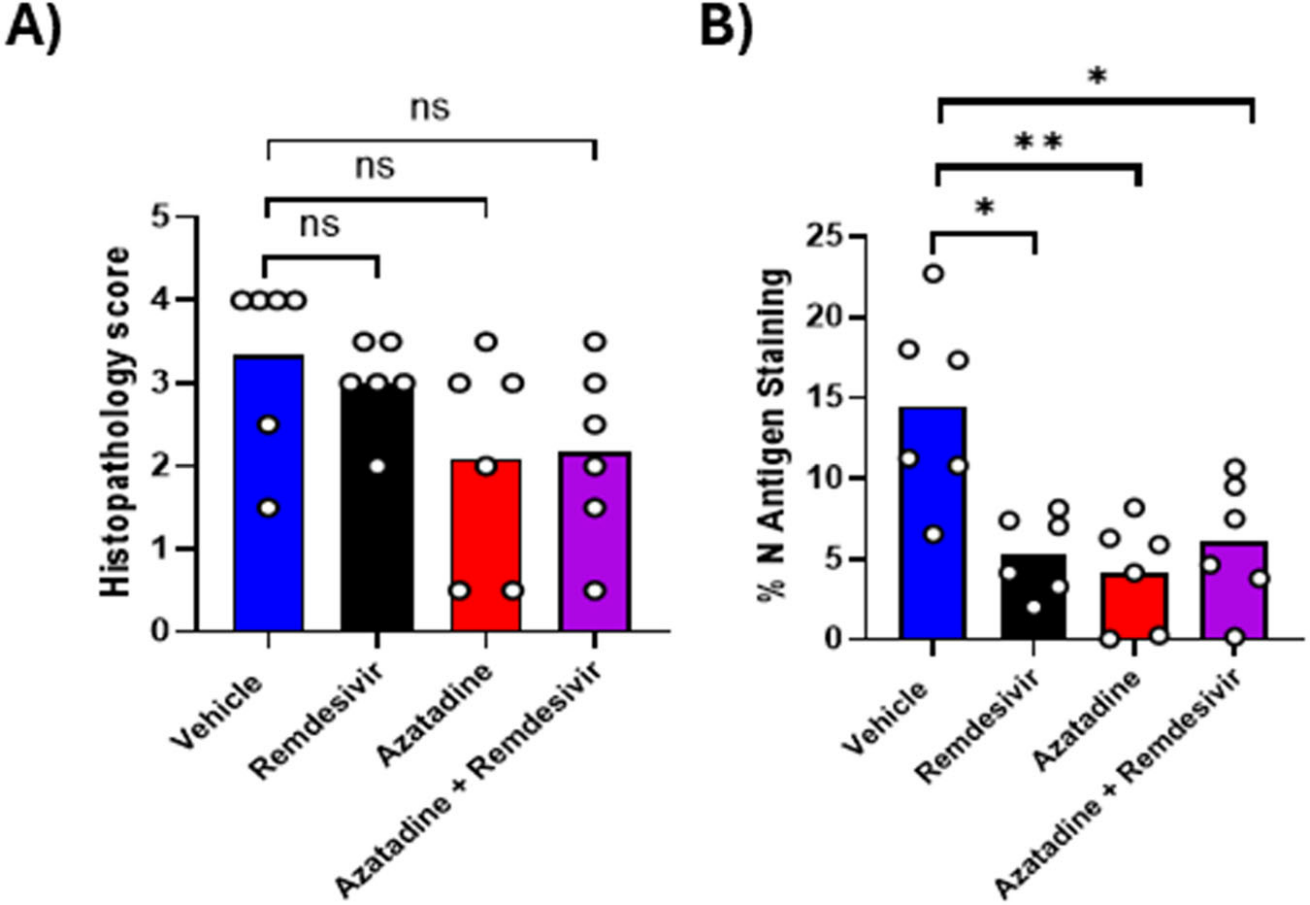
Quantification of histopathological changes and viral antigen expression in the lungs of K18‐hACE2 mice. K18‐hACE2 mice (*n* = 6) were infected intranasally challenged with SARS‐CoV‐2 LIV strain at a dose of 10^4^ PFU SARS‐CoV‐2, and treated with compounds or vehicle alone as indicated, and examined at day 4 post infection. (A) The extent of histopathological changes observed in infected lung tissues were evaluated using a semi‐quantitative scoring system. (B) The amount of SARS‐CoV‐2 N antigen staining was quantified using a morphometric approach, with the percentage of immuno‐stained area (%) expressed as the ratio between the immuno‐stained area and the total area. Data for individual animals are shown with the mean value represented by a column. Side by side comparisons were made using a Mann‐Whitney U test *Represents *p* ≤ 0.05, **represents *p* ≤ 0.01.

Histological changes and viral antigen expression in the lungs were determined using formalin‐fixed, paraffin‐embedded lung tissue specimens. The main changes in all lungs were focal parenchymal areas where alveoli contained desquamated alveolar epithelial cells and leukocytes, together with perivascular lymphocyte‐dominated, predominantly mononuclear infiltrates (Figure. S5). Viral antigen expression was observed in alveolar epithelial cells (both type I and II), generally in variably sized patches of positive alveoli (Figure S5). The inflammatory changes were observed across all experimental groups. They were generally most severe in vehicle controls (Figure S5A; Figure. 8A) and less extensive in treatment groups (Figure S5B‐D), but these differences did not reach statistical significance (Figure 8A). However, viral antigen expression, quantified as the percentage of immuno‐stained area relative to total tissue area, was significantly reduced in all treatment groups (Figure 8B), with Azatadine‐Dimaleate treatment reducing viral antigen expression by approximately 50%. This substantial reduction in viral antigen load provides further evidence of Azatadine‐Dimaleate's potent antiviral activity in vivo.

## 3 Discussion

There are extremely few licensed therapeutics for SARS‐CoV‐2 infection/COVID‐19. While the immediate urgency of the COVID‐19 pandemic has subsided, the need for effective antiviral therapeutics remains compelling as respiratory viruses continue to pose significant global health threats. Drug repurposing has received significant attention as a means to rapidly identify therapeutics as an alternative strategy to conventional drug development. This approach is particularly valuable for pandemic preparedness, enabling rapid deployment of treatments against emerging infectious diseases. Since the beginning of the COVID‐19 pandemic, numerous investigations and clinical trials were initiated to identify therapies for SARS‐CoV‐2. While most failed to demonstrate significant clinical benefits [22], several drug‐repurposing screens have demonstrated encouraging results that deserve further investigation [23, 24, 25, 26, 27].

In this comprehensive study, we applied a high‐throughput screen of over 2,500 drugs to identify inhibitors of SARS‐CoV‐2, followed by validation in multiple model systems, including in vitro cell lines, *ex vivo* human airway epithelial cell cultures and an in vivo K18‐hACE2 transgenic mouse model. Interestingly, we found striking differences in antiviral efficacy of some drugs among the cell lines used. Previous antiviral screens for SARS‐CoV‐2 also highlighted the role of cellular genes in viral infection and cell‐type differences [28, 29, 30]. This underscores the necessity of validating candidates across multiple systems and the importance of using physiologically relevant models for antiviral screening.

In our initial in vitro screening, Azatadine‐Dimaleate demonstrated remarkable antiviral efficacy against SARS‐CoV‐2 with an EC50 of 4.0 µM (95% CI: 3.2–4.8 µM). Notably, amongst 30 antihistamines evaluated, only Azatadine‐Dimaleate exhibited consistent and potent antiviral activity across multiple cell types, suggesting mechanisms beyond canonical H1‐receptor antagonism. This aligns with computational predictions suggesting antihistamines as promising anti‐COVID‐19 candidates [31, 32].

Azatadine‐Dimaleate possesses several distinguishing pharmacological properties that may contribute to its antiviral activity. Unlike many second‐generation antihistamines, Azatadine retains lipophilic properties enabling cellular penetration while maintaining a favourable safety profile [33]. Its tricyclic structure differs from other antihistamines tested, potentially facilitating interactions with viral or host proteins critical for SARS‐CoV‐2 replication. Additionally, Azatadine has documented anti‐inflammatory effects beyond histamine antagonism, including suppression of NF‐κB signalling [34] and reduction of pro‐inflammatory cytokine production [35], which align with our observed reduction in IL‐6 and interferon expression in infected WD‐PBECs. The compound's dual antiviral and anti‐inflammatory properties position it uniquely among COVID‐19 therapeutic candidates, as it simultaneously suppresses viral replication ( > 5,000‐fold reduction at 25 µM) and modulates inflammatory responses.

Indeed, recent preclinical studies on related antihistamines, such as Azelastine, have demonstrated antiviral activity against SARS‐CoV‐2, potentially through interaction with key viral entry components or host factors required for viral replication [36]. *In vitro*, approved H1 inhibitors, such as Ebastine and Mequitazine, inhibited SARS‐CoV‐2 in Vero cells with IC50 values of 6.92 and 7.28 µM, respectively [13]. Loratadine was reported to inhibit SARS‐CoV‐2 with an IC50 of 15.13 µM in Caco‐2 cells [37]. Azelastine, identified in multiple independent screens [38], demonstrated promising clinical translation, with a recent trial by Klussmann and colleagues [39] revealing significant viral load reduction following nasal administration. The therapeutic potential of Azatadine‐Dimaleate is further substantiated by compelling epidemiological evidence. Puigdellívol‐Sánchez and colleagues [40] conducted a comprehensive analysis revealing significantly reduced hospital admission rates among antihistamine users. Similar protective effects were documented by Morán Blanco and colleagues [41] in nursing home residents, and in earlier observational study of 79,083 in Spain, which demonstrated significantly reduced risk among SARS‐CoV‐2 patients receiving H1 receptor antagonists [42].

The demonstrable consistency of Azatadine‐Dimaleate's therapeutic efficacy across progressively complex experimental systems provides compelling evidence of translational potential. A distinctive strength of our investigation lies in validating Azatadine‐Dimaleate's therapeutic efficacy in WD‐PBECs. Our experimental design deliberately incorporated postinfection treatment initiation at 24 h postinfection, authentically modelling the clinical scenario where therapeutic intervention necessarily follows symptom onset. This WD‐PBEC system revealed that Azatadine‐Dimaleate significantly reduced viral titers at 72 and 96 hpi despite intervention during established infection. All treatment regimens maintained viral titers below 10^6^ PFU/mL, contrasting markedly with control cultures which reached 10^7.4^ PFU/mL, representing a clinically relevant 25‐fold reduction in viral replication.

Translation to the in vivo setting provided crucial evidence of therapeutic potential in a complex biological system. In the K18‐hACE2 transgenic mouse model, Azatadine‐Dimaleate‐treated animals exhibited significantly attenuated disease, manifested by reduced weight loss compared to vehicle controls (4% *vs.* 12%, *p* ≤ 0.05). Comprehensive virological assessment revealed decreased viral RNA and infectious virus in pulmonary tissues, accompanied by approximately 50% reduction in viral antigen expression. These findings demonstrated that the therapeutic efficacy of Azatadine‐Dimaleate translates effectively to the in vivo lung environment. Whilst acknowledging certain inherent limitations of the K18‐hACE2 model, including non‐physiological receptor distribution and accelerated disease course [43], this model is useful to assess drug effects on the alveolar epithelium [44]. Indeed, the robust therapeutic effect observed in this stringent challenge model further substantiates the clinical potential of Azatadine‐Dimaleate. Additionally, we acknowledge that formal pharmacokinetic analysis of Azatadine‐Dimaleate tissue distribution, particularly lung exposure following i.p. administration, would strengthen translation of our findings. This represents a limitation of the current study and should be addressed in future work.

The mechanistic basis for Azatadine‐Dimaleate's antiviral activity likely encompasses multiple complementary pathways. The compound possesses well‐established anti‐inflammatory properties, mediated primarily through mast cell stabilization and suppression of leukotriene and pro‐inflammatory cytokine production. This immunomodulatory activity holds particular relevance for COVID‐19, where mast cell activation significantly contributes to pulmonary immunopathological processes through release of inflammatory mediators [45]. The resultant cytokine dysregulation represents a central pathophysiological mechanism underlying severe COVID‐19. The therapeutic potential of mast cell stabilization in mitigating these deleterious inflammatory cascades was previously postulated [46], with H1 receptor antagonism specifically implicated in regulating allergic lung responses and airway inflammation [47]. Beyond these canonical anti‐inflammatory effects, emerging evidence indicates that H1 receptor antagonists may directly inhibit multiple RNA virus infections through distinct mechanisms [48].

The therapeutic relevance of our findings is supported by pharmacokinetic considerations. Standard oral dosing of Azatadine‐Dimaleate (1–2 mg twice daily) achieves peak plasma concentrations of approximately 5–10 µM [49, 50], which exceeds our observed in vitro EC50 of 4.0 µM. This favourable pharmacokinetic profile, combined with Azatadine's excellent oral bioavailability and CNS penetration, suggests that therapeutic concentrations are achievable with approved dosing regimens. However, evidently formal pharmacokinetic/pharmacodynamic modelling in the context of COVID‐19 treatment will be essential for optimizing dosing strategies, and that pharmacokinetic analysis of tissue distribution, particularly lung exposure following i.p. administration in our mouse model, represents a limitation of the current study.

While our study provides compelling evidence for Azatadine‐Dimaleate's antiviral activity, the precise molecular mechanisms remain to be fully elucidated. Future investigations should employ time‐of‐addition experiments to determine whether Azatadine‐Dimaleate acts at viral entry, replication, or assembly stages. Pseudovirus entry assays would definitively establish whether the compound interferes with ACE2 binding or membrane fusion. Additionally, viral resistance selection studies could identify potential target proteins and inform combination therapy strategies.

Importantly, Azatadine‐Dimaleate demonstrated remarkable synergy with Remdesivir (CI = 0.034), substantially enhancing the potency of both compounds. When combined, EC50 values decreased to approximately 0.25 and 2.0 μM for Remdesivir and Azatadine‐Dimaleate, respectively, representing a 2.4‐fold and twofold improvement in antiviral activity. This pronounced synergism likely reflects complementary mechanisms of action; Remdesivir inhibiting viral RNA‐dependent RNA polymerase [51], whilst Azatadine‐Dimaleate exerts both direct antiviral effects and immunomodulatory activities. Such combinatorial approaches offer several potential advantages, including reduced dosing requirements [3], minimizing dose‐dependent adverse effects, and elevating the genetic barrier to resistance development [52]. While our synergy analysis using the Chou‐Talalay method demonstrated strong synergy, comprehensive dose‐matrix studies with isobologram analysis across multiple effect levels would provide more detailed characterization of this interaction.

The clinical translation of Azatadine‐Dimaleate for COVID‐19 treatment could leverage multiple administration routes. While our study employed systemic administration, topical delivery via nasal spray may achieve higher local concentrations at the primary site of infection while minimizing systemic exposure. This approach has shown promise with related antihistamines like Azelastine, which demonstrated viral load reduction in clinical trials following intranasal administration. Combination formulations incorporating both Azatadine‐Dimaleate and Remdesivir could be developed for either systemic or topical delivery, potentially offering enhanced efficacy through their demonstrated synergy.

## 4 Conclusion and Future Directions

As SARS‐CoV‐2 continues its evolutionary trajectory, generating variants with enhanced transmissibility and immune evasion capabilities like FLiRT [53], our comprehensive characterization of Azatadine‐Dimaleate provides a compelling scientific foundation for immediate clinical evaluation. Of particular significance is the potential efficacy against emerging variants exhibiting substantial antigenic shift, such as those observed in the original Omicron lineage and more recently in BA.2.86 derivatives [54, 55]. These antigenically divergent variants represent ongoing challenges to vaccine‐mediated immunity, highlighting the critical necessity for effective antivirals regardless of the pandemic's acute phase resolution. The consistency of efficacy across our multi‐tiered experimental framework positions this compound not merely as a promising candidate for current variants, but as a valuable component of pharmaceutical preparedness against future coronavirus threats. The data presented herein strongly support expedited clinical development of Azatadine‐Dimaleate, either as monotherapy or in strategic combination with Remdesivir, as an essential contribution to global health security infrastructure in this era of unpredictable but inevitable viral emergence.

Future investigations should prioritize elucidation of the precise molecular mechanisms underlying Azatadine‐Dimaleate's antiviral activity, particularly the basis for its distinctive efficacy compared to structurally related antihistamines. Targeted mechanistic studies employing techniques, such as affinity‐based protein profiling, comparative proteomics, and molecular dynamics simulations could identify specific viral or host protein interactions mediating their antiviral effects. Additionally, exploration of alternative administration routes, particularly intranasal delivery, warrants thorough investigation given the localization of initial SARS‐CoV‐2 infection to the respiratory epithelium, similar to the approach successfully demonstrated with Azelastine in human clinical trials [39]. Evaluation of efficacy against emergent SARS‐CoV‐2 variants of concern and related betacoronaviruses would elucidate the breadth of Azatadine‐Dimaleate's antiviral spectrum and its potential utility in future coronavirus outbreaks.

The translation of Azatadine‐Dimaleate into clinical practice offers notable implementation advantages. Its established safety profile could expedite regulatory pathways, whilst existing manufacturing infrastructure enables economical large‐scale production with minimal adaptation requirements. Future studies should specifically evaluate efficacy against a diverse spectrum of antigenically distinct SARS‐CoV‐2 variants to confirm retention of therapeutic potential despite ongoing viral evolution. Furthermore, the compound's stability at ambient temperatures and potential for various administration routes enhances distribution feasibility across diverse healthcare settings, including resource‐limited regions with constrained cold‐chain capabilities, a critical consideration for global pandemic preparedness.

## 5 Materials and Methods

### 5.1 Experimental Design

This study aimed to identify effective repurposed therapeutics against SARS‐CoV‐2 through a multi‐tiered validation approach. Our primary objectives were to screen a diverse library of compounds with established safety profiles, validate promising candidates across systems of increasing physiological complexity, and identify compounds with consistent efficacy predictive of clinical translation potential.

We implemented a systematic three‐tiered validation strategy: high‐throughput screening of 2,570 compounds in Vero and Calu‐3 cell lines, validation in WD‐PBECs, and assessment in the K18‐hACE2 transgenic mouse model. This sequential approach was designed to eliminate compounds with cell‐type specific effects or limited efficacy in physiologically relevant systems.

For screening, compounds were tested at 5 μM with selection criteria of ≥ 80% CPE inhibition and > 90% viability. WD‐PBEC studies employed a postinfection treatment paradigm (24 h postinfection) to model therapeutic intervention. *In vivo* studies used K18‐hACE2 mice treated with compounds before and after challenge. All experiments included appropriate vehicle controls and Remdesivir as a reference standard. *In vitro* experiments were performed in at least triplicate, while animal studies used groups of 6 mice per condition, with endpoints including viral titers, RT‐qPCR quantification, immune response profiles, weight change, and histopathological assessment.

### 5.2 SARS‐CoV‐2 Isolation, Stock Culture and Quantification

All work involving live SARS‐CoV‐2 was conducted in certified Biosafety Level 3 (BSL‐3) facilities at Queen's University Belfast (NI1/20.1) and the University of Liverpool (PP4715265) in compliance with UK Health and Safety Executive regulations and institutional biosafety protocols. Work was performed under appropriate risk assessments and standard operating procedures with staff trained in BSL‐3 practices.

The SARS‐CoV‐2 England/2/2020 (VE6‐T) (EPI_ISL_407073) isolate was acquired from Public Health England, which was designated “‘PHE’.” Another strain was isolated from a patient in Belfast, UK in June 2020. This isolate (isolated on Vero cells) is referred to as BT20.1 [56]. UK strain of SARS‐CoV‐2 (hCoV‐2/human/Liverpool/REMRQ0001/2020; herein called the LIV strain, lineage B), which was cultured from a nasopharyngeal swab from a patient, was used for animal experiments [57]. These virus isolates are described in more detail elsewhere [58]. The PHE stock was passaged 3 times in Vero cells to generate a working stock for drug screening. This working stock carries a deletion of the polybasic cleavage site in Spike, consistent with a previous report [59]. All SARS‐CoV‐2 infections were performed in biosafety level three conditions. Both isolates were expanded to passage 4 on either Vero‐E6 (PHE) or Vero cells (BT20.1). Cells were plated in 175 cm [2] flasks with complete DMEM (5% fetal bovine serum (FBS) without antibiotics) and allowed to attach overnight at 37°C and 5% CO_2_. SARS‐CoV‐2 (MOI = 0.001 to 0.01) was added to infection medium (DMEM with 5% FBS without antibiotics) and the flask was incubated at 37°C and 5% CO_2_ for 4 (PHE) or 3 (BT20.1) days. When maximal CPE was observed, the supernatant was harvested and clarified at 460 x *g* for 5 min. The supernatant was aliquoted and stored at −80°C. Infectious titers were determined by plaque assay as previously described [60]. Vero cells were seeded at 7.5 ×104 cells/well in 24 well plates. The following day virus samples were serially diluted 10‐fold. These dilutions were inoculated on Vero cells for 1 h at 37°C. After adsorption, overlay medium in DMEM was added to give a final concentration of 2% FBS and 0.05% agarose, providing a semi‐solid overlay. At 72 h post infection (hpi), cells were fixed in 4% paraformaldehyde for 30 min and plaques were visualized and counted using Crystal violet (0.1% w/v in methanol).

### 5.3 Cell Line Culture and Infection

Calu‐3 cells, a human lung adenocarcinoma cell line, were obtained from American Type Culture Collection (ATCC) and cultured in MEM, supplemented with 10% (v/v) FBS, 1% (v/v) penicillin/streptomycin, 1% (v/v) l‐glutamine. Vero and Vero‐E6 cells, both *Cercopithecus aethiops*‐derived kidney cell lines, were obtained from ATCC and the MRC‐University of Glasgow, Centre for Virus Research, respectively, and were maintained in DMEM supplemented with 10% (v/v) FBS, 1% (v/v) penicillin/streptomycin. HEK‐293T, BEAS‐2B, A549, and Huh7 cells were obtained from ECACC. HEp‐2 cell were a kind gift from Prof. Ralph Tripp, University of Georgia. Cells were routinely monitored to confirm mycoplasma‐free conditions. All cells were maintained at 37°C and 5% CO_2_. Calu‐3 cells were seeded at a density of 5 × 10^4^ cells/well in 24 well plates. Cells were seeded in the presence of the drugs, with Remdesivir and DMSO used as positive and negative controls, respectively. At 24 h post seeding cells were infected with SARS‐CoV‐2 PHE (MOI = 0.1) and incubated for 72 h. At 72 hpi supernatant was harvested from infected cells and used to determine viral titers by plaque assay. Where RT‐qPCR was performed, RNA was extracted from infected cell cultures.

### 5.4 Drug Libraries

A combined drug library of 2,570 high‐purity compounds ( > 95%) dissolved in high‐quality DMSO were used for the study (Figure. 1‐A). Compound quality control was performed by liquid chromatography‐mass spectrometry and/or ^1^H‐NMR, as detailed by manufacturers. Libraries used were SCREEN‐WELL® FDA approved drug library V2 (700 compounds from Enzo, UK), Antiviral library (350 compounds from MedChemExpress, USA), and virtual Screening Anti‐COVID‐19 compound library based on the 3CL protease (PDB ID: 6LU7), Spike Glycoprotein (PDB ID: 6VSB), NSP15 (PDB ID: 6VWW), RdRp, PLPro and ACE2 (Angiotensin Converting Enzyme 2) structure (1,520 compounds from MedChemExpress, USA). Libraries were prepared at 5 mM, to support a 5 μM screening format. Echo‐qualified 384‐well low dead volume plus microplates (LP‐0200‐BC; Labcyte Inc.) were used as the library source plates to support acoustic transfer with an Echo 525 Liquid Handler (Labcyte Inc.).

### 5.5 SARS‐CoV‐2 High‐Content Screening Assay

Drug compounds were acoustically transferred into black 384 well optical bottom plates (Nunc‐UK). Vero cells (3 × 10^3^ cells/well) or Calu‐3 cells (9 × 10^3^) were seeded in 40 μL medium. The positive control Remdesivir (1 µM) (MedChemExpress‐USA) and the negative control DMSO were spotted on each plate. Following 24 h, plated cells were inoculated with 10 μL SARS‐CoV‐2 diluted in MEM per well at a multiplicity of infection (MOI) of 0.1. CPE was determined 3 days postinfection using CellTox™ Green Cytotoxicity Assay (Promega‐UK), following the manufacturer's instructions. Plates were incubated for 15 min before recording fluorescence (480/520 nm) using a Synergy 2 Multi‐Mode Microplate Reader (CLARIOstar Plus; BMG LABTECH, UK). The screening and validation workflow is summarized in Supporting Figure 3.

### 5.6 Uninfected Host Cell Cytotoxicity Counter Screen

Compounds were acoustically transferred into black 384 well optical bottom plates (Nunc‐UK). Calu‐3 and Vero cells were maintained and seeded as described for the infection assay. Plates were incubated for 72 h at 37°C and 5% CO_2_. To assess cell toxicity, CellTox™ Green Cytotoxicity Assay (Promega‐UK) was undertaken following the manufacturer's instructions. Plates were incubated for 15 min before recording fluorescence (480/520 nm) using a Synergy 2 Multi‐Mode Microplate Reader (CLARIOstar Plus; BMG LABTECH, UK).

### 5.7 Combination Treatments‐ Azatadine‐Dimaleate and Remdesivir

Calu‐3 cells were pretreated 24 h before infection (PHE MOI = 0.1) with either DMSO, Remdesivir (starting at 5 µM), Azatadine‐Dimaleate (starting at 40 µM), or a combination of the two with Remdesivir (starting at 1 µM) and Azatadine‐Dimaleate (starting at 25 µM). In total 6 concentrations of each condition were used.

### 5.8 Real Time Quantitative Polymerase Chain Reaction (RT‐qPCR)

For RNA extraction, cultures were incubated with 400 µL of Lysis/Binding buffer (High Pure Isolation Kit; Roche) for 5 min at room temperature. Total lysed samples were vortexed for 15 s and transferred to a High Pure filter tube and total RNA was extracted using High Pure Isolation Kit (Roche) according to the manufacturer's protocol. RNA was quantified by spectrophotometry and 1 µg was transcribed into cDNA using The High‐Capacity cDNA Reverse Transcription Kit (Applied Biosystems, Thermo Fisher Scientific) in 0.2 mL PCR reaction tubes (Eppendorf). A cDNA reaction mixture containing 10 µL RNA (15 ng/µL) and 10 µL of Master Mix in 1:1 ratio was prepared by running the program in a thermocycler (MJ6 Research PTC‐220 Peltier Thermal Cycler DYAD DNA Engine) for 2 h and 17 min. A primer/probe set for detection of SARS‐CoV‐2 *Nsp12* RNA was developed with the sequences *NSP12*‐Fwd: 5’‐GTGARATGGTCATGTGTGGCGG‐3’, *NSP12*‐Rev: 5’‐CARATGTTAAASACACTATTAGCATA‐3’, and *NSP12*‐P:5’‐FAM‐CAGGTGGAACCTCATCAGGAGATGC‐3’ (Eurofins‐UK). Ready‐made catalogue assays for a set of primers and probes (FAM labelled) (Thermofisher) specific for human genes involved in the innate immune response to SARS‐COV‐2 such as: *IFNL1* (Assay ID: Hs00601677 g1, Cat. No:433118) *IFNB* (Assay ID:Hs01077958_s1, Cat. No. 4331182), *ISG15* (Assay ID: Hs01921425 s1, catalogue no:4331182), *IFIT1* (Hs03027069_s1, Cat no. 4331182), *IL6* (Hs00174131_m1, Cat no. 4331182) and *CXCL8/IL8* (Bt03211906_m1, Cat no. 4331182) were selected for analyses.

When performing qPCR the Roche probe mixes for the detection of *TBP* (Cat. No. 05189284001) and *GAPDH* (Assay ID: Hs02786624_g1, Cat. No:4331182) were used as references. The LightCycler® 480 Probes Master mix was used following the manufacturer's protocol to quantify the mRNA expression levels for all genes of interest. The ΔΔCT method was used to quantify the relative mRNA expression levels of *NSP12* and for all other genes data analysis was performed using the complementary LightCycler 96 software (Roche‐UK). Each experimental condition was evaluated in duplicate and mean relative ratios were plotted.

### 5.9 WD‐PBEC Culture and Infection

Culture and differentiation methods of WD‐PBEC cultures from commercially available (Promocell) donors (Table S1) were previously described [8]. Cells were passed three times in Promocell Airway Epithelial Cell Growth Medium (C‐21160 Promocell) then seeded onto collagen‐coated 6 mm Transwell supports (Corning) at 3 × 10^4^ cells per Transwell. After 4–6 days of submersion, air‐liquid interface (ALI) was initiated by removing the apical medium. Cells were differentiated using PneumaCult ALI medium (Stemcell Technologies). Complete differentiation took at least 21 days. Cultures were only used when hallmarks of excellent differentiation were evident, including extensive cilia coverage and obvious mucus production*^8^*.

WD‐PBECs were infected with SARS‐CoV‐2 at a multiplicity of infection (MOI) = 0.1 by addition of the inoculum on the apical surface and incubation for 1 h at 37°C. This MOI consistently produces robust infection without overwhelming cellular defenses, allowing accurate assessment of antiviral efficacy. Lower MOI experiments (0.1) showed similar inhibition patterns (data not shown). After infection, the inoculum was removed and the apical surface was rinsed once with DMEM (no additives). Apical washes were performed every 24 h thereafter by adding 200 µL DMEM to the apical surface, incubating for 5 min, removing and storing at −80°C.

At 24 hpi WD‐PBECs were treated with 1 µM Remdesivir, 25 µM Azatadine‐Dimaleate, a combination of 0.4 µM Remdesivir and 10 µM Azatadine‐Dimaleate, or DMSO (to equate to final concentration of DMSO in Azatadine‐Dimaleate preparation). Cultures were treated with 500 µL basolaterally and 10 µL apically. Apical treatment was replaced every 24 h following washes. Basolateral treatment was replaced at 48 hpi. Virus titration in apical washes was performed by plaque assay, as described above.

### 5.10 Mice and Virus Infection

Animal work was approved by the local University of Liverpool Animal Welfare and Ethical Review Body and performed under UK Home Office Project Licence PP4715265. Mice carrying the human ACE2 gene under the control of the keratin 18 promoter (K18‐hACE2; formally B6. Cg‐Tg(K18‐ACE2)2Prlmn/J) were purchased from Charles River [10]. Mice were maintained under SPF barrier conditions in individually ventilated cages and provided ad lib pellet food and water.

Animals were randomly assigned into multiple cohorts. K18‐hACE2 mice were treated intraperitoneally (i.p.) with antiviral compounds or vehicle control. Compounds used were: Azatadine‐Dimaleate (10 mg/kg in PBS), remdesivir (in the form of metabolite GS‐441524; 25 mg/kg daily in 10% DMSO, 10% Sulfobutylether‐β‐Cyclodextrin, 80% PBS) [61], a combination of Azatadine‐Dimaleate and remdesivir at the same doses, or vehicle (DMSO). The dose of Azatadine‐ Dimaleate was approximately 8× the ED_50_ of histamine inhibition in the mouse [62] and that of remdesivir was one that was known to be effective against SARS‐CoV‐2 infection in vivo^*20*^.

After 2 h, mice were then challenged with SARS‐CoV‐2 LIV [17] strain via the intranasal route. To do this, mice were anaesthetized lightly with isoflurane and inoculated intranasally with 50 µL containing 10^4^ PFU SARS‐CoV‐2 LIV strain in PBS. Treatment with compounds was repeated every 24 h. The weight of the mice was monitored every 24 h as a noninvasive clinical sign of disease. Four days postinfection, mice were euthanized by an overdose of pentabarbitone and lung tissues collected and analyzed for viral load by qRT‐PCR, virus titers, and histopathological changes and viral antigen expression. Tissues for RNA extraction and virus titration were immediately frozen at ‐80°C until processing.

### 5.11 RNA Extraction From Animal Tissue and qRT‐PCR

The upper lobes of the right lung were dissected and homogenized in 1 mL of TRIzol reagent (Thermofisher‐UK) using a tissue lyser LT (Qiagen) and stainless‐steel beads (Qiagen) at 50 oscillation for 5 min. The homogenates were clarified by centrifugation at 12,000×g for 5 min before full RNA extraction was carried out according to manufacturer's instructions. RNA was quantified and quality assessed using a Nanodrop (Thermofisher‐UK) before a total of 1 μg was DNase treated using the TURBO DNA‐free™ Kit (Thermofisher‐UK) as per manufacturer's instructions. Viral load was quantified using a 1 step RT‐qPCR kit (Promega). For quantification of SARS‐CoV‐2 the nCOV_N1 primer/probe mix from the SARS‐CoV‐2 (2019‐nCoV) CDC qPCR Probe Assay (IDT) were utilized and murine 18 s was used as a housekeeping gene to normalize the qPCR. The 18 s standard was generated by the amplification of a fragment of the murine 18S cDNA using the primers F: ACC TGG TTG ATC CTG CCA GGT AGC and R: AGC CAT TCG CAG TTT TGT AC. Likewise, SARS‐CoV‐2 genomic N1 standard was generated by PCR using the qPCR primers. cDNA was generated using 1 step qRT‐PCR kit (Promega) as per manufacturer's instructions. Both PCR products were purified using the QIAquick PCR Purification Kit (Qiagen) and serially diluted 10‐fold from 10^10^ to 10^4^ copies/reaction to form the standard curve.

### 5.12 Histology, Immunohistology, and Morphometric Analysis

The left lung was fixed in 10% neutral buffered formalin for 48 h, then stored in 70% ethanol until processing and in its entirety routine paraffin wax embedded. Consecutive sections (3–‐4 µm) were either stained with hematoxylin and eosin (HE) or used for immunohistology to detect viral antigen expression, using the horseradish peroxidase (HRP) method and rabbit anti‐SARS‐CoV nucleocapsid protein (Rockland, 200–‐402‐A50) as previously described [11].

The histopathological changes in the lungs were semi‐quantitatively scored based on the presence and size of areas with desquamated alveolar epithelial cells and leukocytes in alveolar lumina (+, ++, +++) and the presence and extent of perivascular lymphocyte‐dominated infiltrates (+, ++, +++); the two scores were added to a final score.

For quantification of viral nucleocapsid protein (NP) expression in the lung, a morphometric analysis was undertaken on the slides stained for SARS‐CoV‐2 NP, as previously described [20]. The stained lung sections were scanned (NanoZoomer 2.0‐HT; Hamamatsu, Hamamatsu City, Japan) and analyzed using the Visiopharm 2022.01.3.12053 software (Visiopharm, Hoersholm, Denmark) to quantify the area that showed immunostaining for SARS‐CoV‐2 NP in the lung of all infected animals. In Visiopharm, for each section, the lung was manually outlined and annotated as Region Of Interest (ROI), manually excluding artefactually altered areas. The manual tissue selection was further refined with an Automated Analysis Protocol (APP) based on a Decision Forest classifier, with the pixels from the Regions of Interest (ROI) being ultimately classified as either “Tissue” or “Background”. This new “Tissue” ROI was further quantified by executing two APPs successively. The first APP was based on a Threshold classifier and served to detect and outline areas with immunostaining. The second APP then measured both the surface of the immunostained area (µm^2^) and the surface of the “Tissue” ROI (µm^2^). The percentage of immunostained area (%), expressed as the ratio between the immunostained area and the total area, was obtained for each animal in Excel (Microsoft Office 2019; Microsoft, Redmond, Washington, United States), according to the following formula: ([positive area (µm^2^)]/[total area (µm^2^)]) × 100.

### 5.13 Statistical Analysis

For the drug screening assay, each assay plate contained one column of control wells with mock infected cells, and one column of control wells with cells infected with SARS‐CoV‐2. All control wells were treated with DMSO at the same concentration as assay wells and used to calculate a Z'‐scores for each plate and to normalize the data on a per plate basis. Results were expressed as percent inhibition of CPE, where 100% inhibition of CPE was equal to the mean of the mock infected cell controls, and 0% of inhibition was equal to the mean of the SARS‐CoV‐2 infected cell controls.

For RT‐qPCR, relative ratios for each gene of interest were measured in relation to the reference gene (*GAPDH* or *TBP*) using a LightCycler 96 software (Roche). Unpaired Student's t‐test was used to calculate statistical significance between DMSO control and drug treatments individually using Graph‐pad Prism Version 9.1.

Combination Index (CI) values were calculated using the CompuSyn Software (ComboSyn Inc) based on the Chou‐Talalay method for drug combination analysis [63]. This quantitative approach defines additive, synergistic, and antagonistic effects using the equation CI = (D)₁/(Dx)₁ + (D)₂/(Dx)₂ + α[(D)₁(D)₂]/[(Dx)₁(Dx)₂], where (D)₁ and (D)₂ represent the concentrations of Azatadine‐Dimaleate and Remdesivir in combination, whilst (Dx)₁ and (Dx)₂ are the concentrations of individual drugs producing the same effect. CI values ≤ 0.9 indicates synergy; CI values 0.9–‐1.1 indicates an additive effect and CI > 1.1 indicates antagonism.

For the animal experiments, sample sizes were determined by a priori power calculations using G*Power software with parameters set to detect a 30% difference in viral load reduction and weight loss between treatment and control groups (power=0.8, α = 0.05), yielding a required sample size of n = 6 animals per group. Weight change over time was analyzed using two‐way ANOVA with Bonferroni correction for multiple comparisons. Viral loads and morphometric measurements were compared using one‐way ANOVA with Dunnett's multiple comparison test or Mann‐Whitney U test as appropriate. Statistical significance was defined as *p* ≤ 0.05.

## Author Contributions

Ahlam Ali and Ultan F. Power conceived and designed the overall study. Ahlam Ali, David Courtney, Lindsay Broadbent conducted the experiments. Parul Sharma, A.K., E.B, A. Ki, J.Parul Sharma, Ultan F. Power, designed the animal studies and Parul Sharma, Eleanor Bentley, A.K., performed them. Ahlam Ali, Ken I. Mills, J.Parul Sharma, A. Ki., Ultan F. Power analyzed data. Ahlam Ali wrote the manuscript with input from all authors. All authors reviewed and approved the final manuscript.

## Conflicts of Interest

Several of the authors (AA, DC, UP, KM, KB, CB, OT) are inventors on a patent (WO 2022/195296 A1) describing the use of azatadine as a single or combination therapy for COVID‐19 patients.

## Supporting information


**Fig. S1:** Screening of a panel of human and simian cell lines, including HEK‐293T, BEAS‐2B, A549, HEp‐2, Huh7, Vero, and Vero E6 cells to identify cells that are permissive to SARS‐CoV‐2 infection. Cells were seeded in 24 well plates. The following day, virus samples were serially diluted 10‐fold. After adsorption overlay medium was added providing a semi‐solid overlay. At 72 hpi, cells were fixed in 4% paraformaldehyde for 30 min and plaques were visualized and counted using Crystal violet solution (0.1% w/v in methanol).

**Fig. S2:** A) The composition of the repurposing library to include 700 FDA approved drugs, 350 Antiviral drugs, and 1520 virtual Screening Anti‐COVID‐19 compounds based on 3CL protease, Spike Glycoprotein, NSP15, RDRP, PLPro and ACE2 structure. B) Drug screen workflow: compounds were pre‐spotted in 384‐well plates at a final concentration of 5 µM, followed by cell seeding and 24 h incubation before infection with a clinical isolate of SARS‐CoV‐2 at an MOI of 0.1. CPE induced by the virus was measured using CellTox™ Green Cytotoxicity Assay. C) Drug screen protocol validation using 5 µM Remdesivir as a positive control and DMSO as a negative control. The graph shows Log_2_ fold change of cytotoxicity levels after normalization to the median of each plate for all positive and negative controls, as well as for non‐infected cells, across all screening plates. D) The correlation plot of fold change fluorescence of drug compounds in the two replicates. R^2^ indicates the correlation coefficient for the replicates.

**Fig. S3:** Compound attrition funnel illustrating the systematic screening and validation cascade for SARS‐CoV‐2 antiviral discovery. The schematic depicts the progressive selection process from initial high‐throughput screening to final lead identification. Starting from 2,570 compounds screened in duplicate using CPE‐based assays in both Vero and Calu‐3 cells, 53 compounds (2.1%) demonstrated > 80% inhibition of virus‐induced cytopathic effect and were identified as primary hits. Of these, 40 compounds were selected for detailed dose‐response validation based on potency in primary screens and activity across both cell lines. Secondary validation using seven‐point dose‐response curves identified 12 compounds with reproducible antiviral activity. Stringent selection criteria requiring EC50 values < 10 µM with acceptable safety profiles yielded 3 validated candidates: Azatadine‐Dimaleate (EC50 = 4.0 µM), Lycorine hydrochloride (EC50 = 0.67 µM), and GS‐441524 (EC50 = 0.85 µM). Azatadine‐Dimaleate was prioritized for further development based on its novel mechanism among antihistamines, favorable safety profile as an FDA‐approved drug, and demonstrated synergy with Remdesivir.

**Fig. S4:** Determination of safety profile of 2,570 drugs in Calu3 (A) and Vero cells (B). Drugs were added at a concentration of 5 µM and cells were incubated for 72 h. Cytotoxicity was measured using CellTox™ Green reagent in the Clariostar plus plate reader. All control wells were treated with DMSO at the same concentration as assay wells. Results were expressed as cell toxicity normalized relative to controls. Each data point represents an average of two separate repeat experiments.

**Fig. S5:** Histological changes and viral antigen expression in the lungs of K18‐hACE2 mice after intranasal challenge with 10^4 PFU SARS‐CoV‐2 LIV, euthanised at 4 dpi. All lungs show parenchymal areas with desquamated alveolar epithelial cells and leukocytes in alveoli and perivascular lymphocyte dominated, predominantly mononuclear leukocyte infiltration (HE stain; left column), with generally widespread alveolar epithelial cell infection (immunohistology for viral NP expression; right column). (A) Vehicle treated animal; histopathology score 4. There are large areas with desquamated alveolar epithelial cells and leukocytes in alveoli (asterisk; bottom inset: arrowhead) as well as a moderate perivascular leukocyte infiltration (top inset), combined with extensive alveolar viral antigen expression. (B) Remdesivir treated animal; histopathology score 3. There are small random areas with desquamated alveolar epithelial cells and leukocytes in alveoli (bottom inset: arrowhead) as well as a moderate perivascular leukocyte infiltration (top inset), combined with large focal areas of alveolar viral antigen expression. (C) Azatadine‐ Dimaleate treated animal; histopathology score 2. There are small random areas with desquamated alveolar epithelial cells and leukocytes in alveoli (bottom inset: arrowhead) as well as a mild perivascular leukocyte infiltration (top inset: arrowhead), combined with multifocal areas of alveolar viral antigen expression. (D) Azatadine‐Dimaleate/Remdesivir treated animal; histopathology score 2. There are small random areas with desquamated alveolar epithelial cells and leukocytes in alveoli (bottom inset: arrowhead) as well as a mild perivascular leukocyte infiltration (top inset: arrowhead), combined with large focal areas of alveolar viral antigen expression. Bars = 1 mm and 25 µm (insets).

**Table S1:** Percentage of CPE inhibition following primary screening of 40 selected drugs in both Vero and Calu‐3 cell lines. The table includes targets and clinical information about the drugs. Drugs highlighted in white, grey and blue were effective, respectively, in both cell lines, in Calu‐3 cells only, or in Vero only cells, based on ≥ 80% CPE inhibition.

**Table S2.:** Percentage of CPE inhibition for drug candidates in Calu3 (A) and Vero cells (B).

## Data Availability

All data supporting the findings of this study are available within the manuscript and its supplementary information files. Raw screening data and additional datasets are available from the corresponding author upon reasonable request. The data that support the findings of this study are available from the corresponding author upon reasonable request.
