# Supplementary figures and images for: Preclinical Screening Platform Identifies Azatadine‐Dimaleate as a Potent Repurposed Therapeutic Against SARS‐CoV‐2 Infection
Source: J Med Virol. 2025 Nov 20;97(11):e70713. doi: 10.1002/jmv.70713 (PMC12631721; doi:10.1002/jmv.70713)

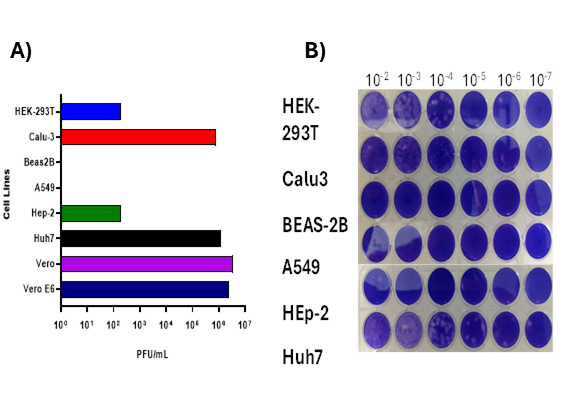

Supplement: Supplementary file 1 — Fig. S1: Screening of a panel of human and simian cell lines, including HEK‐293T, BEAS‐2B, A549, HEp‐2, Huh7, Vero, and Vero E6 cells to identify cells that are permissive to SARS‐CoV‐2 infection. Cells were seeded in 24 well plates. The following day, virus samples were serially diluted 10‐fold. After adsorption overlay medium was added providing a semi‐solid overlay. At 72 hpi, cells were fixed in 4% paraformaldehyde for 30 min and plaques were visualized and counted using Crystal violet solution (0.1% w/v in methanol). [file JMV-97-e70713-s007.tif]

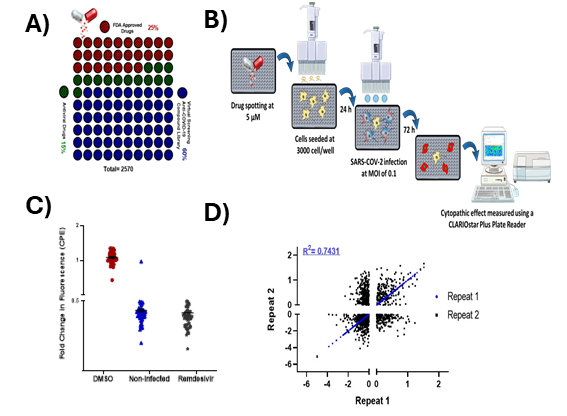

Supplement: Supplementary file 2 — Fig. S2: A) The composition of the repurposing library to include 700 FDA approved drugs, 350 Antiviral drugs, and 1520 virtual Screening Anti‐COVID‐19 compounds based on 3CL protease, Spike Glycoprotein, NSP15, RDRP, PLPro and ACE2 structure. B) Drug screen workflow: compounds were pre‐spotted in 384‐well plates at a final concentration of 5 µM, followed by cell seeding and 24 h incubation before infection with a clinical isolate of SARS‐CoV‐2 at an MOI of 0.1. CPE induced by the virus was measured using CellTox™ Green Cytotoxicity Assay. C) Drug screen protocol validation using 5 µM Remdesivir as a positive control and DMSO as a negative control. The graph shows Log2 fold change of cytotoxicity levels after normalization to the median of each plate for all positive and negative controls, as well as for non‐infected cells, across all screening plates. D) The correlation plot of fold change fluorescence of drug compounds in the two replicates. R2 indicates the correlation coefficient for the replicates. [file JMV-97-e70713-s002.tif]

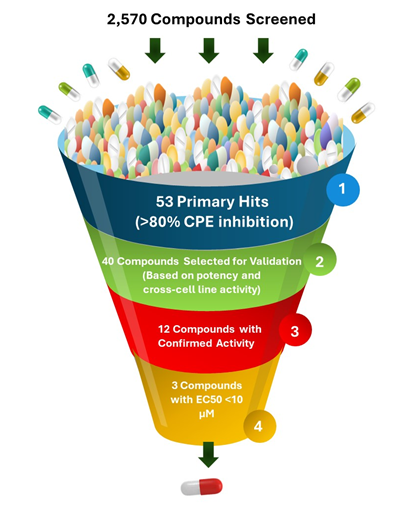

Supplement: Supplementary file 3 — Fig. S3: Compound attrition funnel illustrating the systematic screening and validation cascade for SARS‐CoV‐2 antiviral discovery. The schematic depicts the progressive selection process from initial high‐throughput screening to final lead identification. Starting from 2,570 compounds screened in duplicate using CPE‐based assays in both Vero and Calu‐3 cells, 53 compounds (2.1%) demonstrated > 80% inhibition of virus‐induced cytopathic effect and were identified as primary hits. Of these, 40 compounds were selected for detailed dose‐response validation based on potency in primary screens and activity across both cell lines. Secondary validation using seven‐point dose‐response curves identified 12 compounds with reproducible antiviral activity. Stringent selection criteria requiring EC50 values < 10 µM with acceptable safety profiles yielded 3 validated candidates: Azatadine‐Dimaleate (EC50 = 4.0 µM), Lycorine hydrochloride (EC50 = 0.67 µM), and GS‐441524 (EC50 = 0.85 µM). Azatadine‐Dimaleate was prioritized for further development based on its novel mechanism among antihistamines, favorable safety profile as an FDA‐approved drug, and demonstrated synergy with Remdesivir. [file JMV-97-e70713-s006.tif]

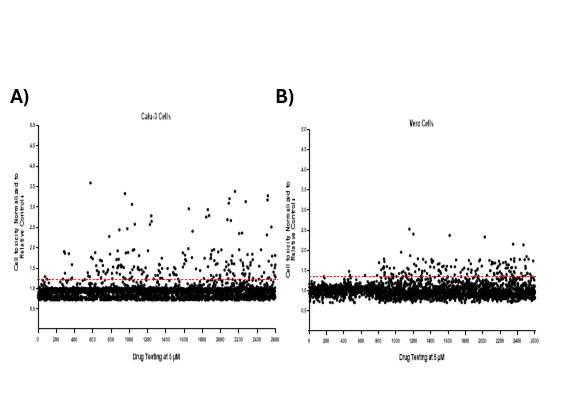

Supplement: Supplementary file 4 — Fig. S4: Determination of safety profile of 2,570 drugs in Calu3 (A) and Vero cells (B). Drugs were added at a concentration of 5 µM and cells were incubated for 72 h. Cytotoxicity was measured using CellTox™ Green reagent in the Clariostar plus plate reader. All control wells were treated with DMSO at the same concentration as assay wells. Results were expressed as cell toxicity normalized relative to controls. Each data point represents an average of two separate repeat experiments. [file JMV-97-e70713-s005.tif]

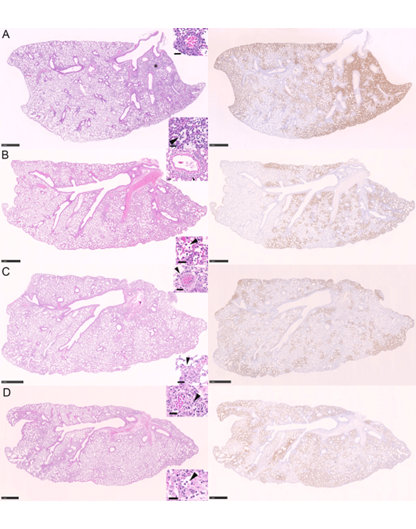

Supplement: Supplementary file 5 — Fig. S5: Histological changes and viral antigen expression in the lungs of K18‐hACE2 mice after intranasal challenge with 10^4 PFU SARS‐CoV‐2 LIV, euthanised at 4 dpi. All lungs show parenchymal areas with desquamated alveolar epithelial cells and leukocytes in alveoli and perivascular lymphocyte dominated, predominantly mononuclear leukocyte infiltration (HE stain; left column), with generally widespread alveolar epithelial cell infection (immunohistology for viral NP expression; right column). (A) Vehicle treated animal; histopathology score 4. There are large areas with desquamated alveolar epithelial cells and leukocytes in alveoli (asterisk; bottom inset: arrowhead) as well as a moderate perivascular leukocyte infiltration (top inset), combined with extensive alveolar viral antigen expression. (B) Remdesivir treated animal; histopathology score 3. There are small random areas with desquamated alveolar epithelial cells and leukocytes in alveoli (bottom inset: arrowhead) as well as a moderate perivascular leukocyte infiltration (top inset), combined with large focal areas of alveolar viral antigen expression. (C) Azatadine‐ Dimaleate treated animal; histopathology score 2. There are small random areas with desquamated alveolar epithelial cells and leukocytes in alveoli (bottom inset: arrowhead) as well as a mild perivascular leukocyte infiltration (top inset: arrowhead), combined with multifocal areas of alveolar viral antigen expression. (D) Azatadine‐Dimaleate/Remdesivir treated animal; histopathology score 2. There are small random areas with desquamated alveolar epithelial cells and leukocytes in alveoli (bottom inset: arrowhead) as well as a mild perivascular leukocyte infiltration (top inset: arrowhead), combined with large focal areas of alveolar viral antigen expression. Bars = 1 mm and 25 µm (insets). [file JMV-97-e70713-s003.tif]

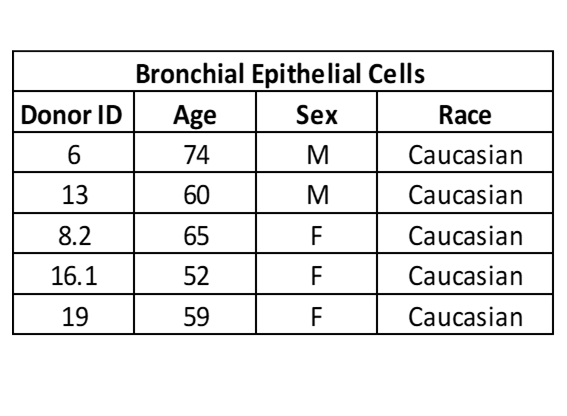

Supplement: Supplementary file 6 — Table S1: Percentage of CPE inhibition following primary screening of 40 selected drugs in both Vero and Calu‐3 cell lines. The table includes targets and clinical information about the drugs. Drugs highlighted in white, grey and blue were effective, respectively, in both cell lines, in Calu‐3 cells only, or in Vero only cells, based on ≥ 80% CPE inhibition. [file JMV-97-e70713-s004.tif]

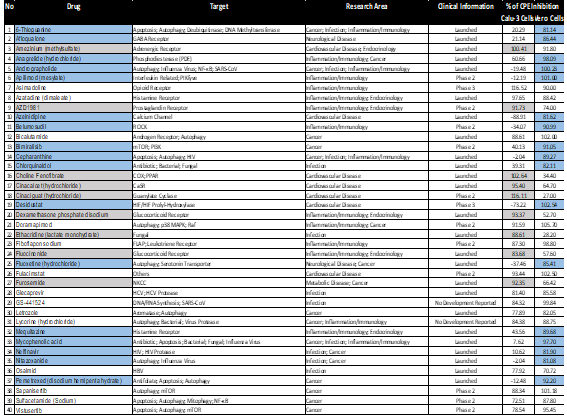

Supplement: Supplementary file 7 — Table S2.: Percentage of CPE inhibition for drug candidates in Calu3 (A) and Vero cells (B). [file JMV-97-e70713-s001.tif]
